# Dispersal strategies shape persistence and evolution of human gut bacteria

**DOI:** 10.1016/j.chom.2021.05.008

**Published:** 2021-07-14

**Authors:** Falk Hildebrand, Toni I. Gossmann, Clémence Frioux, Ezgi Özkurt, Pernille Neve Myers, Pamela Ferretti, Michael Kuhn, Mohammad Bahram, Henrik Bjørn Nielsen, Peer Bork

**Affiliations:** 1 Gut Microbes and Health, Quadram Institute Bioscience, NR4 7UQ Norwich, UK; 2 Digital Biology, Earlham Institute, NR4 7UZ Norwich, UK; 3 European Molecular Biology Laboratory, Structural and Computational Biology Unit, 69117 Heidelberg, Germany; 4 Department of Animal Behaviour, Bielefeld University, Bielefeld DE-33501, Germany; 5 Inria, INRAE, CNRS, Univ. Bordeaux, 33405 Talence, France; 6 Clinical Microbiomics A/S, Copenhagen, Denmark; 7 Department of Biotechnology and Biomedicine, Technical University of Denmark, DK-2800 Kgs. Lyngby, Denmark; 8 Department of Ecology, Swedish University of Agricultural Sciences, Ulls väg 16, 750 07 Uppsala, Sweden; 9 Institute of Ecology and Earth Sciences, University of Tartu, Vanemuise 46, 51014 Tartu, Estonia; 10 Max Delbrück Center for Molecular Medicine, Berlin, Germany; 11 Yonsei Frontier Lab (YFL), Yonsei University, Seoul 03722, South Korea; 12 Department of Bioinformatics, Biocenter, University of Würzburg, Würzburg, Germany

**Keywords:** metagenomics, population genetics, gut microbiome, strain resolution, bacterial dispersal, antibiotics

## Abstract

Human gut bacterial strains can co-exist with their hosts for decades, but little is known about how these microbes persist and disperse, and evolve thereby. Here, we examined these processes in 5,278 adult and infant fecal metagenomes, longitudinally sampled in individuals and families. Our analyses revealed that a subset of gut species is extremely persistent in individuals, families, and geographic regions, represented often by locally successful strains of the phylum Bacteroidota. These “tenacious” bacteria show high levels of genetic adaptation to the human host but a high probability of loss upon antibiotic interventions. By contrast, heredipersistent bacteria, notably Firmicutes, often rely on dispersal strategies with weak phylogeographic patterns but strong family transmissions, likely related to sporulation. These analyses describe how different dispersal strategies can lead to the long-term persistence of human gut microbes with implications for gut flora modulations.

## Highlights

• Bacterial strains may persist within family members through transfer
• Bacteria adapt dispersal strategies: heredipersistent, spatiopersistent, and tenacious
• Dispersal strategies correlate with genetic bottlenecks and effective population size

## Graphical abstract

**Figure:**
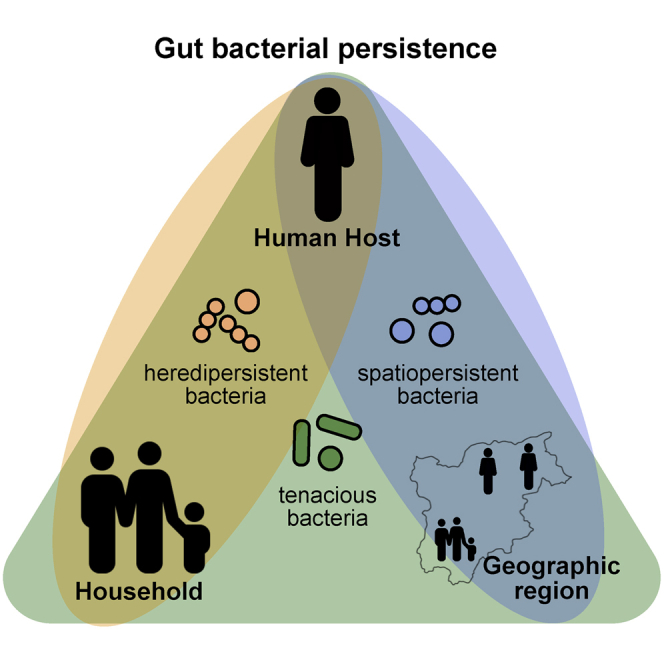

## Teaser

Hildebrand et al., investigated strategies of bacterial persistence using publicly available time series metagenomes from stool samples. They found that three major dispersal strategies underlie human gut bacterial persistence. These have distinct evolutionary patterns such as the accumulation of rare variation and differences in their strength of selection.

## Introduction

Bacterial persistence is the continued occurrence of a bacterial strain and its clonal offspring. In the human gut, bacterial strains can persist for many years (Faith et al., 2013; Nielsen et al., 2014; Schloissnig et al., 2013). This relationship is likely the result of a co-evolved symbiosis between microbes and their host, providing (predictable) benefits to both partners over longer times (Dethlefsen et al., 2007; Moran et al., 2019). Therefore, understanding persistence is important for comprehending human health and wellbeing. However, the scales and mechanisms of persistence remain largely unexplored: strains could persist either because of their own traits or enabled by the host (Foster et al., 2017). Gut bacteria may predominantly colonize hosts vertically, received early in life from a parent and persisting thereafter. Alternatively, bacteria could also persist due to frequent recolonizations of a host, persistence enabled through frequent reintroductions. Thus, dispersal strategy, bacterial transmission, and colonization of new environments may all contribute to persistence. Disentangling these processes can help us understand which bacteria need protection, are restorable, or are likely to be replaced over time.

Human gut strain colonization and replacement are likely shaped by a combination of strain adaptation and survival in the host as well as the influx of better-adapted strains, which is particularly dynamic during early life (Ferretti et al., 2018; Vatanen et al., 2016; Yassour et al., 2016). Colonizing bacteria might ingress from family members (Brito et al., 2019; Korpela et al., 2018; Xie et al., 2016) as well as environmental sources (reviewed in [Browne et al., 2017]). Dispersal abilities and geographic distribution range can vary between bacterial species. For example, strains of the gastrointestinal symbiont *Helicobacter pylori* are limited in their geographic dispersal with a strong phylogeographic signal (Montano et al., 2015), and are often transmitted in host families (Didelot et al., 2013). On a reduced scale, limited geographic dispersal has been reported for gut bacterial subspecies (Costea et al., 2017; Truong et al., 2017). Thus, the scale of strain dispersal could differ vastly between our gut bacteria, ranging from strictly vertical (parent-child) transmissions, to the global occurrence of strains and their dispersal following a model of “everything is everywhere, but the environment selects” (Baas-Becking, 1934). Yet, it remains largely unclear how the dynamic and frequent host colonization by gut bacterial strains is, and how it may depend on the host environment.

Here, we investigated gut bacterial persistence in relation to dispersal at the individual, family, and geographic levels. We hypothesize that different bacterial phyla might have profoundly different strategies to ensure their continued persistence and evolution.

## Results

### Metagenomic strategy and dataset

To encompass as many individuals and nationalities as possible, we compiled a dataset of 5,278 fecal metagenomes, mostly longitudinally sampled. 290 samples were from an unpublished family cohort and combined with samples from 20 published studies (STAR Methods). These samples represented 2,089 individual hosts (average 2.6 samples/individual, maximum 41). The average observation time was 131 days, and 242 individuals were observed for ≥365 days, and 33 individuals were observed for ≥1,000 days (Figures S1A and S1C). In total, 417 individuals were part of a total of 191 healthy families. The data covered different age groups, including adults (>18 years of age, 56%) and children (defined as ≤3 years of age, 41%). Fecal microbiota transfers related samples (n = 59), were excluded from all analysis, except genome binning.

**Figure 1:**
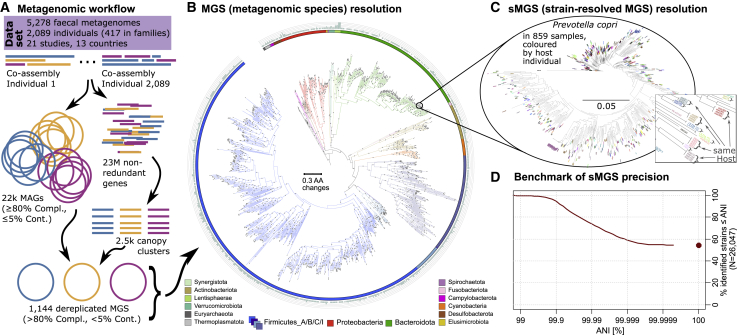
Bioinformatic workflow leading to strain-resolved metagenomic species (A) 5,278 longitudinal metagenomes were co-assembled per individual host (n = 2,089) (Figure S1). From these co-assemblies, a gene catalog with 23,137,742 genes was created and used to cluster 2,474 canopies. In parallel, metagenomic assembled genomes (MAGs) were calculated from the co-assemblies. MAGs and canopy clusters were combined and dereplicated to 1,144 high quality (>80% completeness, <5% contamination) MGS. (B) Phylogeny and taxonomic assignment of all 1,144 MGS. The outer circle indicates missing taxonomic assignment levels (species, genus, family, order, and class), all MGS had at least phylum-level assignments, 83% were named at the genus level. Branches with >90 bootstrap support have gray circles. (C) Intraspecific phylogeny exemplified for *Prevotella copri*. 859 sMGS were reconstructed from 859 metagenomic samples with ≥2X *P*. *copri* coverage, tree tips are randomly colored by the host individual. Monophyletic sMGS within the same host or host family were used to identify strains persisting in individuals or families. (D) Identified strains were used to benchmark sMGS precision. The average nucleotide identity (ANI) was calculated between genetic sequences of strains found recurrently in individuals or families, using core genes of a species (see STAR Methods). 55% of these sequences were completely identical (100% ANI), with 95% of strains having <99.9% ANI in their representative sequences. For brevity, MGS and sMGS will be referred to as species and strains, respectively, in the main text. MAG, metagenomic assembled genome; compl., Cont, completeness and contamination of genomic bin; MGS, metagenomic species; sMGS, strain-delineated MGS; ANI, average nucleotide identity.

All metagenomic samples (n = 5,278) were co-assembled per individual, genes predicted on these assemblies and a non-redundant gene catalog were constructed from these genes. Both metagenomic assemblies and a gene catalog were used to bin representative genomes of the dominant bacteria across all gut samples, resulting in 1,144 high-quality genomes of metagenomic species (MGS). After controlling for frequently occurring species, 440 of 1,144 MGS were retained for strain-resolved analysis using the here-developed concept of strain-resolved MGS (sMGS, see Figure 1 for workflow and estimated strain resolution). Tracking sMGS allowed us to calculate persistence in longitudinal samples, family transmissions, phylogeographic patterns, and evolutionary genome statistics (Table 1; STAR Methods).

**Table 1:** Terminology used throughout the manuscript to define different forms of host-bacterial association at differing spatial scales

	Primary	Secondary	Measure
Tenacity (bacterial persistence within a host, family and geographic region)	persistence	strain persistence	percent of observed time spanned by longitudinal samples with identical strain
		strain resilience	fraction of consecutive longitudinal samples harboring identical strain
		annual persistence	annual strain survival (Kaplan-Meier analysis)
	family association	horizontal strain transmission (parent-parent)	fraction of identical strains in host families, where species were present in pairwise samples
		vertical strain transmission (child-parent)	
	phylogeography	country association	within-country compared with between-country strain phylo. dist. (perMANOVA)
		geographic associations	correlation of strain phylo. dist. to geographic distance of samples (Mantel)

Identical strains were defined as groups of monophyletic strains in intraspecific phylogenies. PerMANOVA, permuted multivariate analysis test; Mantel, Mantel test for comparing two distance matrices; phylo. dist, phylogenetic distance based on intraspecific phylogeny.

### Gut bacterial persistence is widespread but heterogeneous between taxa

Gut bacterial persistence in a single human host was investigated by tracking bacterial strains in longitudinal samples. A strong average persistence was observed, exemplified by 277 of 440 gut species having a survival chance of 100% after one year. The mean strain persistence, resilience, and annual persistence were 91%, 84%, and 95%, respectively (Table 1). This corresponds well to similar analyses from literature, e.g., 60% of gut strains persisted for ≥5 years based on culturing and 16S rRNA amplicon sequencing (Faith et al., 2013) and an average 1-year retention of 90% of strains was found for 35 gut species (Table S13 in [Schloissnig et al., 2013]).

Persistence differed between prokaryotic phyla. Bacteroidota had a very high strain persistence (mean 99%) as did Euryarchaeota (99%, representing genus *Methanobrevibacter*), and Cyanobacteria (99%, representing the family *Gastranaerophilaceae*). Strain persistence was low for Firmicutes_A (91%), followed by Actinobacteriota (94%), and Proteobacteria (96%) (Figure S1S; Table S1). When considering the average persistence for bacterial genera, a similar pattern emerged: within Bacteroidota, the genera such as *Prevotella*, *Alistipes*, and *Parabacteroida* had a high strain persistence, in contrast to a generally low persistence in the Firmicutes genera. The genera *Veillonella*, *Escherichia*, *Enterococcus*, and *Enterobacter* had relatively lower persistence rates (Figure S1), possibly indicating a relatively higher turnover rate in the gut environment. As expected, strain persistence strongly correlated to a species’ occurrence (p < 1e–30, Spearman correlation test), but not to relative species abundance (p = 0.4, accounting only for samples with a species’ presence).

Persistence differences between taxa likely depend on traits encoded in the bacterial genomes. For example, (Browne et al., 2017) found sporulation capacity and oxygen tolerance were negatively correlated to persistence. In line with this, we found a negative correlation of a species’ persistence with the number of sporulation and oxygen tolerance genes in their genomes (p < 2e–7). Although the occurrence of these genes varied systematically between bacterial phyla (e.g., Firmicutes were enriched in sporulation genes), this correlation remained significant within most phyla separately (Figures S2A and S2B).

### Bacterial persistence differs with host age, delivery mode, and antibiotic usage

**Figure 2:**
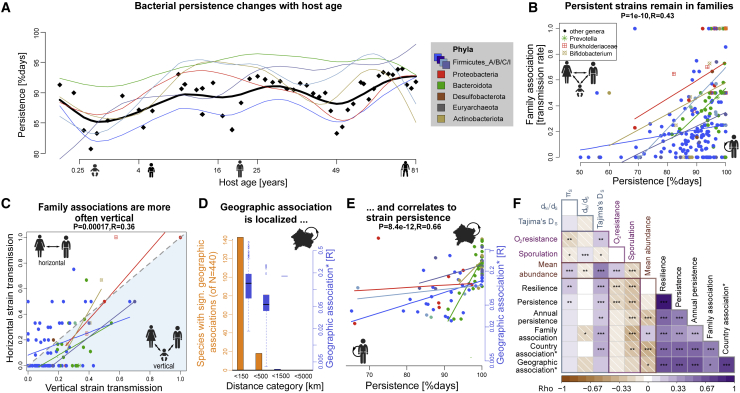
Gut bacterial persistence extends beyond the individual host association (A) Strain persistence consistently increased with host age. The black line is the average, colored lines the six most abundant phyla. Average persistence was highest in Bacteroidota strains, especially in infants (green line). Dots are the average values in each age window, lines are smoothed splines of data points. Each individual host is represented as their median age. See Figure S2 for delineation of antibiotic exposed hosts. The same taxa colors are used in all panels unless otherwise noted. (B) Species that are persistent in an individual have a higher probability of being transmitted within a family. (C) The frequency of vertical transmission in families (parent-child, n = 203 pairs) was often higher than horizontal transmission (parent-parent, n = 13 pairs). Species with <2 potential transmissions (total, vertically and horizontally, and arbitrary threshold) were excluded. (D) For most of the 440 microbial species, geographic associations were only significant at a local scale (<150 km, 142/440 species, orange bars). The strength of geographic association decreased on average at higher distances (measured as the correlation coefficient between genetic and geographic distance, blue boxplots). Boxplot centers represent the median; the edges represent first and third quartiles. (E) Persistence and geographic association (across all distance classes, only significant values included) were highly correlated; Bacteroidota (green) and Actinobacteriota (ochre) were notable for their steep correlations. Only species with significant geographic associations were included. (F) Correlogram of the most important population genetic parameters (synonymous nucleotide diversity [π_S_], excess of rare alleles [Tajima’s D_s_ at synonymous sites], non-synonymous to synonymous substitutions [d_N_/d_S_]), and how they correlate with different forms of bacterial persistence, family, and phylogeography as well as species’ mean abundance. Stars denote multiple testing corrected Spearman correlation tests: ^∗^q < 0.05, ^∗∗^q < 0.01, ^∗∗^q < 0.001. Only species with significant country or geographic associations were included in correlations involving these.

While our analyses have so far shown that taxonomy and gene content play a crucial role in gut bacterial persistence, it is unclear whether other factors may also be involved. To address this, we investigated several intrinsic host features as possible determinants of bacterial persistence. When comparing host ages, bacterial persistence was lower in children (mean 80% strain persistence; 76% strain resilience, Table 1) than in adults (mean 93% strain persistence; 85% strain resilience). This was expected as in newborn infants, bacterial strains are often exchanged in the first months of life (Ferretti et al., 2018; Korpela et al., 2018). However, species able to persist in adults were also more likely to persist in infants, as there was a correlation between adults’ and infants’ strain persistence (p = 3e–4, R = 0.25). Relating strain persistence and resilience to host age showed that host association increased during host aging, being lowest in infants and highest in adults (Figures 2A and S2D). This was also true when separating persistence by phyla: Bacteroidota were more persistent than other phyla, especially in infants. Actinobacteriota (including *Bifidobacterium*) had low strain persistence, particularly in infants, possibly related to weaning (Kujawska et al., 2020). While taxonomic composition varied between infants and adults, the microbiome was gradually enriched for “persistent” species until the age of 10 years, a pattern that was observed in different phyla (Figures S2E and S2F). Thus, it seems that the aging microbiome is increasingly colonized by species that persist, reaching a steady state after 10 years.

In addition to age, we had information on birth mode and antibiotic usage in several studies included in this meta-analysis. In children born via C-section (n = 27), average strain persistence was 98% compared with 87% in those vaginally born (n = 295). This 8-fold increase in odds ratio (OR) affected the most prevalent phyla (p < 1e–16, Fisher’s exact test). However, strain resilience was not different for these children, fitting to initial colonizing strains being retained longer in C-section born infants (Podlesny and Fricke, 2021). Antibiotic exposure lowered mean strain resilience from 85% to 80% (n = 143 individuals exposed to antibiotics, n = 1,355 not exposed). This detrimental effect of antibiotics was highly significant among the phyla Bacteroidota and Firmicutes_A (p < 1e–16, OR 0.7) and observed at various host ages (Figure S2E). However, strain persistence was only reduced from a mean of 93% to 91% upon exposure to antibiotics (p = 0.003, OR = 0.88, Fisher’s exact test). Indeed, a significantly higher persistence for *Desulfovibrionaceae*, *Lachnospiraceae*, *Lactobacialles*, *Bifidobacterium*, *Faecalibacterium*, and Enterobacteriaceae was observed, while *Prevotella* and *Bacteroides*, among other genera, were reduced in their persistence (Table S2A). These disjunct results between persistence and resilience reflect the latter measuring strain replacements. A resilient strain is unlikely to become even “less replaced” than the baseline resilience, while persistence, the time a strain is observed, might be positively influenced by antibiotic interventions.

### Linked persistence and family transmissions of gut bacteria

The family association score, representing transmission of strains between family members, correlated with persistence (p = 1e–10, R = 0.43, Figure 2B), resilience (p = 1e–15, R = 0.51), and annual persistence (p = 1e–4, R = 0.26, Table 1). Investigating family association at the genus level, *Sutterella*, *Duodenibacillus* (Proteobacteria), and *Dialister* (Firmicutes_C) had strong family associations (Figure S1; Tables S2B and S2C). From our dataset, we determined six high-confidence, highly transmitted species (having >20 paired observations between hosts of the same family, and family associations >50%). These included: *Bacteroides stercoris*, *Bacteroides massiliensis*, *Holdemanella biformis*, *Sutterella wadsworthensis*, and *Bifidobacterium bifidum* (with the overall highest family transmission rate at 72%). Only detected in seven host pairs at sufficient abundance, but still noteworthy was *Methanobrevibacter smithii*: this archaeon had a 100% family transmission rate but was only observable at single time points in children (it might not have persisted for longer or was undetectable).

To investigate whether gut bacteria are predominantly colonizing their host between generations, we distinguished vertically (parent-infant pairs, n = 203) and horizontally (parent-parent pairs, n = 13, excluding siblings) shared strains, differentiating colonization resistance of conspecific strains in established (horizontal) and new (vertical) microbiomes. Overall, vertical and horizontal transmission of bacteria were correlated (p = 2e–4, R = 0.36, Figure 2C), suggesting that successful commensals can colonize a new host at any age. However, infant colonization could be the predominant dispersal mode, as the horizontal was usually half of the vertical transmission rate (mean OR was 0.5, exceptions were Proteobacteria and Desulfobacterota). Bacteroidales was the bacterial class with the highest vertical transmission rate, having on average a 2.5 higher chance of being vertically shared in proximal hosts (OR = 0.4, p = 3e–4, Fisher’s exact test, Table S2B).

### Persistent bacteria are dispersed at a local scale by blooming strains

At least for some gut bacteria, geographic dispersion is limited (Costea et al., 2017; Truong et al., 2017), as reflected in a species’ phylogeographic pattern. In our data with a larger sample set, we found that 22% of 440 species had significant geographic associations (p < 0.05, Q < 0.1, Mantel test of geographic to genetic distance, Table 1). Yet, this is likely an underestimate as these p values were negatively correlated to strains observed per species (R = −0.45). Indeed, 49% of gut species had a significant phylogeographic pattern, using the complimentary “country association” (p < 0.05, Q < 0.1, permuted multivariate analysis of variance [perMANOVA] test of within to between-country genetic distance), explaining on average 7% of intraspecific genetic variation. The results from these approaches agreed mostly as 81% of significant phylogeographic associations overlapped.

To better understand gut bacterial phylogeography, we investigated cases of strong geographic associations. For example, 13/14 Italian individuals were colonized by monophyletic strains of *Rothia mucilaginosa*, while in *Muribaculaceae* sp., US and Israeli strains were different to Finish, Russian, Estonian, and Kazakhstan strains. *Odoribacter laneus* was almost exclusively represented by a single, monophyletic clade in US samples (several independent studies), and one *Prevotella* sp. was represented by a monophyletic group found only in Kazakhstan (Figures S3A and S3B). These examples indicate that we often observed the expansion of an especially successful monophyletic clade on a local scale. Indeed, phylogeographic patterns were usually only significant in the lowest tested distance class (<150 km, Figure 2D). But if local strain expansions are driving phylogeographic patterns, we would expect imbalanced phylogenetic trees. Indeed, we found geographic association correlating negatively to median phylogenetic tree distance (p = 6e–6, R = −0.47), but correlating positively to the normalized Sackin’s index, measuring imbalances in phylogenetic trees (p = 7e–6, R = 0.47, Figures S3C and S3D). Thus, species with stronger phylogeographic patterns had imbalanced phylogenies and expansions of select lineages, typically observed in species undergoing local expansions (Dearlove and Frost, 2015).

Both geographic and country associations were positively correlated to persistence, resilience, and annual persistence (p < 1e–10, R ≥ 0.59, Figures S3E and S3F), independently of age and antibiotic exposure. Also, family association correlated with both measures of phylogeography (p < 0.01, R > 0.4, Figures S3G and S3H). This relation was stronger for Bacteroidota and Actinobacteriota species, although persistence in all phyla correlated positively to phylogeography (Figure 2E). Genus *Prevotella* was noteworthy for its overall extreme host associations, having strong family associations, persistence, and geographic associations (mean 0.65, 0.99, and 0.4, respectively). This makes sense in a model where *Prevotella* strains are long-term associated and prevalent in an individual or family, enabling the observed regionally successful strains.

### Comparing persistence, family association, and phylogeography reveal bacterial dispersal strategies

**Figure 3:**
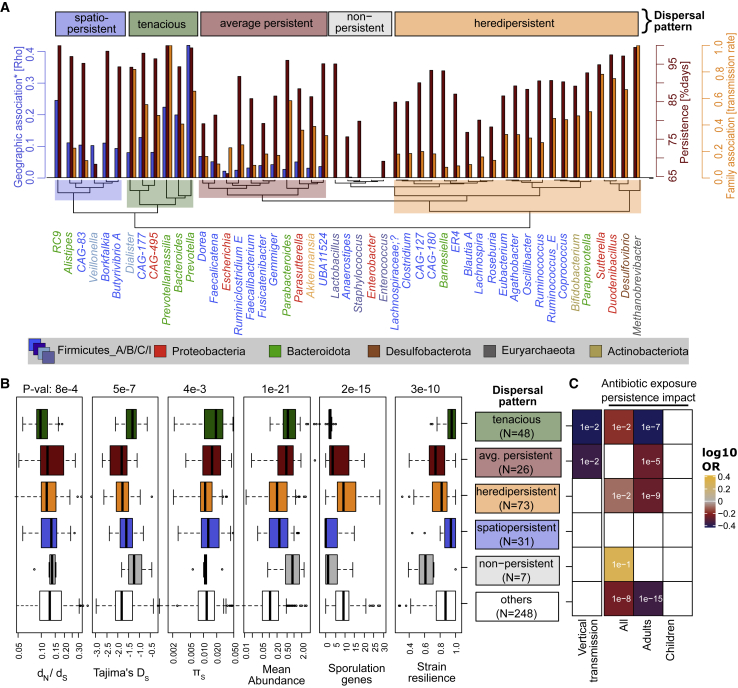
Dispersal patterns of gut bacteria and their link to bacterial evolution (A) Dispersal patterns of bacteria were reflected in their associations: strong geographic and host association (spatiopersistent); strong family and host association (heredipersistent); no associations (non-persistent); all associations (tenacious); and average geographic or family and host associations (average persistent). The analysis was restricted to 50 high abundant genera. (B) Tenacious taxa could be genetically well-adapted (high purifying selection [d_N_/d_S_], fewer selective sweeps [Tajima’s D_s_], high population sizes [π_S_]), while non-persistent taxa show opposing population genetics. p values are calculated with a non-parametric Kruskal-Wallis test, comparing all six groups. (C) Tenacious bacteria are significantly more often transmitted vertically than horizontally in families, having a higher likelihood to be inherited between generations. Antibiotic usage usually reduces strain persistence, especially in adult hosts and tenacious bacteria. The color reflects the log_10_ OR (odds ratio), the value in the squares is the rounded, multiple testing corrected q value of Fisher’s exact test conducted separately for each square, q ≥ 0.1 are shown as white squares. Children are hosts < 3 years old. d_N_/d_S_, non-synonymous to synonymous nucleotide substitutions.; π_S_, synonymous nucleotide diversity; OR, odds ratio in Fisher’s exact test. Boxplot centers represent the median; the edges represent first and third quartiles.

Imperfect correlations between persistence, family associations, and phylogeography imply that there could be an underlying structure in dispersal strategies. To test this, we clustered association measures for the 50 most abundant genera (Figure 3A), revealing several dispersal patterns: (1) *tenacious* bacteria with high phylogeography, family associations, and persistence, (2) *spatiopersistent* bacteria with strong phylogeography and persistence but no family associations, and (3) *heredipersistent* bacteria with strong family associations and persistence but lacking notable phylogeographic signals, as well as (4) average persistent and (5) non-persistent bacteria.

Heredipersistence implies the ability to spread beyond a single host, for instance due to sporulation capacity or oxygen tolerance (Browne et al., 2017); indeed, heredipersistent species were enriched for sporulation genes (Figures 3A and 3B). While most heredipersistent species belonged to the phylum Firmicutes, species from other phyla were also represented such as Proteobacteria (*Duodenibacillus*, *Sutterella*), *Verrucomicrobiota (Akkermansia)*, Archaea (*Methanobrevibacter*), Desulfobacterota_A (*Desulfovibrio*), and Actinobacteriota (*Bifidobacterium*). For this dispersal pattern, strain resilience was noticeably reduced, indicating higher strain turnover rates (Figure S4C). This fits with the lack of phylogeographic signals in this group, implying a wide geographic distribution range for its members.

The most distinct group were tenacious species that excelled in all forms of associations. Tenacity was often observed in the phylum Bacteroidota (e.g., *Prevotella*, *Bacteroides*, and *Prevotellamassilia*) but also *Dialister*, CAG495, and CAG117 (phyla Firmicutes and Proteobacteria). The already outstanding persistence and resilience of tenacious bacteria were even further increased in infants, compared with other bacteria (Figure S4). In combination with a significantly increased vertical transmission (Figure 3C), these microbes could disperse preferentially by inheritance between host generations. However, tenacious bacteria’s persistence and resilience were also most impacted by antibiotic exposure (Figure 3C).

### Dispersal strategies relate to bacterial evolution

We hypothesized that species that are widespread without geographic association, such as heredipersistent bacteria, should show a genomic signature of frequent re-colonization of novel environments. Mechanisms facilitating such frequent gut colonizations should lead to intra-species competition and, if selection is a major mechanism underlying colonization, to signatures of selective sweeps (Smith and Haigh, 1974). This might pose additional challenges to gut commensals, as bacterial life in the gut could be already dominated by population bottlenecks and selective sweeps: several times a day, new food sources can be colonized by competing bacterial strains, only to be expelled upon host defecation, a classical “boom-and-bust” demography. In line with this, the genetic signatures of strong selective sweeps can be found in gut bacteria (Garud et al., 2019; Ramiro et al., 2020; Zhao et al., 2019). Population genetics theory predicts that in populations that are recovering from selective sweeps or population bottlenecks, an excess of rare polymorphisms caused by recent mutations can be observed, resulting in negative Tajima’s D values (Tajima, 1989).

We calculated Tajima’s D_s_ on metagenomic strain genotypes, using synonymous sites to reduce the effects of selection. Tajima’s D_s_ was negative for all 440 species (mean −1.7). In particular, Firmicutes_A species had low Tajima’s D_s_ values (mean −1.8), including important taxa such as *Faecalibacterium*. This might be related to sporulation, as Tajima’s D_s_ negatively correlated to the number of sporulation genes found in a genome, but not to oxygen resistance genes (Figure 2F). Tajima’s D_s_ was higher for Bacteroidota, Proteobacteria, Firmicutes_I (mean −1.35), exemplified in the genera *Bacteroides*, *Akkermansia*, *Lactobacillus*, and *Enterobacter* (mean Tajima’s D_s_ −0.9, Figure S4). In accordance with the highly persistent or even tenacious characteristics of the latter taxa, Tajima’s D_s_ significantly correlated with strain persistence as well as family, geographic, and country associations (p = 8e−6, 8e−6, 0.02, and 4e−5, respectively, all R ≥ 0.22, Figure 2F).

These data imply that taxa strongly associated with host, family or geographic region undergo fewer bottlenecks and show signatures of selection as a consequence of adaptation to individual hosts or families. Indeed, tenacious bacteria had the highest levels of purifying selection among dispersal patterns, estimated through the ratio of non-synonymous to synonymous nucleotide substitutions, d_N_/d_S_ (Figure 3B). Further, selection might be more effective in tenacious taxa. This is because the effectiveness of selection increases with effective population size (Ne). Ne correlates to synonymous nucleotide diversity, π_S_, if mutation rates are equal among species (Charlesworth, 2009). π_S_, and implied Ne, was highest in tenacious bacteria, so was the consensus population size (Nc), estimated by mean relative abundance observed in metagenomes (Figure 3B). These population genetic estimates, therefore, indicate that tenacious bacteria are highly adapted to the human host.

## Discussion

By analyzing gut bacterial strain biogeography at three levels (human host, household, and region), our study provides a view into bacterial dispersal strategies and how they shape the persistence of the gut microbiome. The large cohort size enabled us to describe strong correlations among bacterial persistence, family association, and phylogeography. These connections seem intuitive—persistent bacteria in an individual have more chances to colonize proximal host family members—but to the best of our knowledge, this had not been previously demonstrated. Clustering these variables allowed us to identify gut bacterial dispersal strategies, enabling us to describe tenacious, heredipersistent, and spatiopersistent bacteria associated with individuals, families, and regions to different degrees.

It is possible that only a few bacterial species are host-adapted and have the metabolic flexibility, to persist within the human gut during drastic ecosystem changes such as weaning and the development of the immune system. Tenacious bacteria best fit these requirements, having relatively high vertical-to-horizontal dispersal rates with the highest strain persistence in infants (Figures 3 and S4). This finding is not unexpected, as typical tenacious taxa such as *Bacteroides* spp. are enriched in vertical transmissions in infants (Nayfach et al., 2016; Yassour et al., 2018), their absence is even linked to the abnormal development of the infant microbiome (Shao et al., 2019). The broad carbohydrate metabolism observed in Bacteroidota (Kaoutari et al., 2013) can reflect metabolic flexibility to changing the host diet. Lacking sporulation genes is likely a key to this dispersal pattern, ensuring persistence and phylogeography by avoiding excessive intraspecific competition. Tenacious commensals have high effective and consensus population sizes (Ne and Nc), evolving under purifying (negative) selection (d_N_/d_S_) and relatively fewer population bottlenecks (Tajima’s D_s_, Figure 3C). Such a genetic pattern might emerge due to early, long-term host colonizations with extreme selection for well-adapted commensals and successful association with an individual. We would therefore expect that tenacious bacteria could develop (1) local adaptations to individuals, families, geographic regions, and (2) have a narrower host spectrum than heredipersistent taxa.

Spatiopersistent species had similar characteristics to tenacious bacteria but the crucial difference was their reduced strain persistence in infants (Figure S4). Similarly, persistence of heredipersistent taxa was low in infants, suggesting active ingression and selection of conspecific strains during the first decade of life. The presence of sporulation genes, absence of phylogeographic patterns, and strong genetic bottlenecks observed for these commensals support such a dispersal, reminiscent of the hypothesis “everything is everywhere, but the environment selects” (Baas-Becking, 1934). Accordingly, we observed higher strain replacement rates and a broader occurrence of microbes with the heredipersistent dispersal strategy. It seems likely that most of the replaced strains also colonize close family members, given the strong family associations of heredipersistent gut microbes. And although heredipersistent dispersal of gut bacteria among family members was characteristic to Firmicutes strains, the highest family associations were observed for other taxa (e.g. *Methanobrevibacter*, *Bifidobacterium*, and *Sutterella* and its relative *Duodenibacillus*).

We conclude that heredipersistent microbes could owe their persistence partly to reinfections of their host from external sources. Therefore, these strains might get slowly replaced when the host is living in a different environment or through constant exposure via probiotics. However, their replacement through singular medical interventions (such as fecal microbiota transplants) might be a futile effort—in contrast to tenacious and spatiopersistent bacteria that are more likely permanently replaceable. It is therefore not surprising that tenacious bacteria and their ability to persist are the most negatively affected by antibiotic exposure. But it is also alarming to us because these strains could potentially stem from our childhood and parents, and be likewise important for the next generation. We propose that the maintenance and manipulation of the human gut microbiome must be re-evaluated based on bacterial dispersal strategies because these will affect the persistence and resilience of both existing and newly introduced gut microbes.

## STAR★Methods

### Key resources table

**Table:** 

REAGENT or RESOURCE	SOURCE	IDENTIFIER
**Biological samples**		

Metagenomic sequences of longitudinal samples	This paper	SRA: PRJEB41102

**Deposited data**		

Gene catalogue nucleotide and amino acid sequences	This paper	http://vm-lux.embl.de/∼hildebra/Drama_GC
Kushugulova et al., 2018	https://www.ebi.ac.uk/ena/browser/home	SRA: PRJEB17632
Willmann et al., 2015	https://www.ebi.ac.uk/ena/browser/home	SRA: PRJEB10391
Bäckhed et al., 2015	https://www.ebi.ac.uk/ena/browser/home	SRA: PRJEB6456
Chu et al., 2017	https://www.ebi.ac.uk/ena/browser/home	SRA: PRJNA322188
Asnicar et al., 2017	https://www.ebi.ac.uk/ena/browser/home	SRA: PRJNA339914
Lee et al., 2017	https://www.ebi.ac.uk/ena/browser/home	SRA: PRJNA353655
Heintz-Buschart et al., 2016	https://www.ebi.ac.uk/ena/browser/home	SRA: PRJNA289586
Human Microbiome Project Consortium, 2012	HMP webpage	N/A
Kostic et al., 2015	https://diabimmune-17.ltdk.helsinki.fi/	N/A
Yassour et al., 2016	https://www.ebi.ac.uk/ena/browser/home	SRA: PRJNA290381
Vatanen et al., 2016	https://www.ebi.ac.uk/ena/browser/home	SRA: PRJNA290380
Ferretti et al., 2018	https://www.ebi.ac.uk/ena/browser/home	SRA: PRJNA352475
Mehta et al., 2018	https://www.ebi.ac.uk/ena/browser/home	SRA: PRJNA354235
Zeevi et al., 2015	https://www.ebi.ac.uk/ena/browser/home	SRA: PRJEB11532
Bengtsson-Palme et al., 2015	https://www.ebi.ac.uk/ena/browser/home	SRA: PRJEB7369
Raymond et al., 2016	https://www.ebi.ac.uk/ena/browser/home	SRA: PRJEB8094
Palleja et al., 2018	https://www.ebi.ac.uk/ena/browser/home	SRA: ERP022986
Yassour et al., 2018	https://www.ebi.ac.uk/ena/browser/home	SRA: PRJNA475246
Scholz et al., 2016, Ward et al., 2016	https://www.ebi.ac.uk/ena/browser/home	SRA: PRJNA63661
Li et al., 2016	https://www.ebi.ac.uk/ena/browser/home	SRA: PRJEB12357

**Software and algorithms**		

R version 3.6.2	R Core Team 2017	https://www.r-project.org/
Rarefaction scripts	Saary et al., 2017	https://github.com/hildebra/Rarefaction
Shotgun metagenomic data processing pipeline	Hildebrand et al., 2019	https://github.com/hildebra/MATAFILER
Read depth windows calculation	Hildebrand et al., 2019	https://github.com/hildebra/rdCover
Frameshifts fixing in MSAs program	This paper	https://github.com/hildebra/MSAfix
MetaBAT2 version 2.15	Kang et al., 2019	https://bitbucket.org/berkeleylab/metabat/src/master/
sdm version 1.47	Hildebrand et al. 2014	https://github.com/hildebra/sdm
MegaHit version 1.2.3 beta	Li et al. 2015	https://github.com/voutcn/megahit
Prodigal version 2.6.1	Hyatt et al., 2010	https://github.com/hyattpd/Prodigal
Bowtie2 version 2.3.4.1	Langmead and Salzberg, 2012	https://github.com/BenLangmead/bowtie2
Samtools version 1.3.1	Li et al., 2009	http://www.htslib.org/
BedTools version 2.21.0	Quinlan and Hall, 2010	https://github.com/arq5x/bedtools2
CD-HIT version 4.6.1	Fu et al., 2012	http://weizhongli-lab.org/cd-hit/
MMseqs2 Version f5a1cdb44c9 96d6be229226b09ecc687646c0c12	Steinegger and Söding, 2017	https://github.com/soedinglab/MMseqs2
CheckM version 1.0.11	Parks et al., 2015	https://ecogenomics.github.io/CheckM/
MAFFT version 7.245	Katoh and Standley, 2013	https://mafft.cbrc.jp/alignment/software/
Trimal version 1.4.rev22	Capella-Gutiérrez et al., 2009	http://trimal.cgenomics.org/
IQ-TREE version 1.6.3a	Nguyen et al., 2015	https://github.com/iqtree/iqtree2
iTOL	Letunic and Bork, 2016	https://itol.embl.de/
GTDB-TK version 1.3.0	Parks et al., 2018	https://github.com/Ecogenomics/GTDBTk
Kraken2 version 2.0.9-beta	Wood et al., 2019	http://ccb.jhu.edu/software/kraken2/
Lambda version 1.9.3	Hauswedell et al., 2014	https://seqan.github.io/lambda/
EggNOG-mapper version 2.0.0	Huerta-Cepas et al., 2019	https://github.com/eggnogdb/eggnog-mapper
Diamond version 0.9.24.125	Buchfink et al., 2015	https://github.com/bbuchfink/diamond
Bcftools mpileup version 1.9	Li, 2011	https://samtools.github.io/bcftools/howtos/variant-calling.html
Vegan R package	Oksanen et al., 2014	https://cran.r-project.org/web/packages/vegan/index.html
FUBAR	Murrell et al., 2013	http://www.hyphy.org/
APE	Paradis et al., 2004	https://cran.r-project.org/web/packages/ape/index.html

### Resource availability

#### Lead contact

Further information and requests for resources and reagents should be directed to and will be fulfilled by the Lead Contact, Falk Hildebrand (Falk.Hildebrand@quadram.ac.uk).

#### Materials availability

This study did not generate new unique reagents.

#### Data and code availability

##### Data availability

Metagenomics sequences from 290 samples are deposited in the European Bioinformatics Institute-Sequence Read Archive (SRA) database under accession SRA: PRJEB41102. A list of all public metagenomic studies used and their accession numbers is provided at http://vm-lux.embl.de/∼hildebra/Drama_GC. The gene catalogue nucleotide and amino acid sequences, aggregate metadata, and study lists have been deposited at http://vm-lux.embl.de/∼hildebra/Drama_GC.

##### Code availability

The C++ program to rarefy matrices is available under https://github.com/hildebra/Rarefaction. The pipeline to process shotgun metagenomic samples is available under https://github.com/hildebra/MATAFILER. The C++ program to calculate read depth windows is available under https://github.com/hildebra/rdCover. The C++ program to fix frameshifts in MSAs is available under https://github.com/hildebra/MSAfix.

### Experimental model and subject details

#### Cohort

To create a comprehensive cohort, we reviewed literature for metagenomic studies that sampled participants longitudinally. We downloaded metagenomic DNA sequencing data from 20 studies focused on longitudinal sampling for at least part of the study (Asnicar et al., 2017; Bäckhed et al., 2015; Bengtsson-Palme et al., 2015; Chu et al., 2017; Ferretti et al., 2018; Heintz-Buschart et al., 2016; Human Microbiome Project Consortium, 2012; Kostic et al., 2015; Kushugulova et al., 2018; Lee et al., 2017; Li et al., 2016; Mehta et al., 2018; Palleja et al., 2018; Raymond et al., 2016; Scholz et al., 2016; Vatanen et al., 2016; Ward et al., 2016; Willmann et al., 2015; Yassour et al., 2016, 2018; Zeevi et al., 2015). Metadata were obtained from the supplementary information attached to each publication, or from the authors upon email request. Since in (Bäckhed et al., 2015) the age of mothers could not be shared, we simulated it based on median (31) and quantiles (28-35) assuming a Gaussian distribution.

This was supplemented by 290 samples from our laboratory, N=132 samples were published previously in (Costea et al., 2017; Hildebrand et al., 2019), as noted in Table S3B. This Heidelberg centered cohort was created within the my.microbes project (my.microbes.eu/) (Voigt et al., 2015). The study adheres to the WMA Declaration of Helsinki and was approved by EMBL Bioethics Internal Advisory Board. In total, 36 individuals were included, their average age was 25.5 years old, 32% of samples and 52% of individuals were female. Including family cohorts, 13 individuals we <20 years old, one individual was a newborn baby (Table S3C).

The cohort, sample numbers, mean age and female % per cohort are described in Table S3A. All aggregated metadata from this project is available in Table S3B, totalling to 5,278 metagenomic samples with 2,089 individuals.

### Method details

#### Sequencing of fecal samples

Fecal samples were collected and conserved under anearobic conditions, short term stored at -20°C and subsequently kept for long term storage at -80°C. Genomic DNA was extracted from frozen fecal samples using the GNOME© DNA Isolation Kit (MP Biomedicals). For newly sequenced samples (Table S3B), the Illumina HiSeq 4000 (Illumina, San Diego, CA, USA) platform was used for Library generation and random shotgun sequencing of the fecal samples. All samples were paired-end sequenced with 150 bp read lengths at the Genomics Core Facility, European Molecular Biology Laboratory, Heidelberg, to an approximated sequencing depth of 10 Gbp per sample (Table S3B).

#### A framework for strain-resolved metagenomics without the need for reference genomes

Metagenomic data processing was implemented in the MATAFILER pipeline, previously used to bin and track the genome of a candidate species in a time series (Hildebrand et al., 2019). For each unique host individual, metagenomic samples were co-assembled. Genes predicted on these assemblies were clustered into a new gene catalogue with 23,137,742 genes (95% similarity cut-off). Two complementary approaches were used to bin bacterial genomes: metaBat2 (Kang et al., 2019) and canopy clustering (Nielsen et al., 2014). A central gene catalogue was used to link MAGs to co-occurring genes, remove genes inconsistently linked to a MGS and to track genes within each species across the thousands of samples. This integrated approach offers several advantages compared with relying solely on MAGs: MGS delineate inherently species at 95% nucleotide identity and allowed us to improve binning statistics for MGS (average completeness/contamination for MAGs was 91.9%/1.1%, and for MGS was 93.2%/0.7%, respectively, for bins with >80% completeness, <5% contamination). MGS also provide a set of tractable core genes that are MGS specific (see below for details; Figure 1C; STAR Methods). 1,144 species-level MGS (metagenomic species) were derived, predicted to be ≥80% complete and ≤5% contaminated (Figures 1A and 1B), for brevity referred to as “species” in the main text. In this framework, MGS are represented by groups of genes that are frequently observed in the same genome bin, thereby a bias to only retain the species’ core genome is inherent to our method. The retrieved core genome allowed us to track species consistently across thousands of samples.

To resolve strains, we introduce strain-resolved metagenomic species (sMGS) to delineate MGS into highly resolved genotypes of a MGS, found in the different metagenomes. sMGS were reconstructed for each MGS, based on SNV (single nucleotide variants) in single-copy core genes (average 66±28 genes/MGS) for each metagenomic sample with sufficient read coverage (>2x). The genetic differences between sMGS core genes were then used to calculate an intra-MGS phylogeny, each sMGS representing the MGS’ genotype in our metagenomic samples (examples in Figure 1C). Conspecific strains in the same metagenome cannot be resolved using this method, but we excluded samples that seemed to harbour conspecific sMGS’ (see Supplemental information). Because the aim of the project was to describe strain persistence, the analysis is restricted to sMGS present in ≥ 30 samples (arbitrarily chosen cut-off, Figure S1B). These MGS represents the majority of all sMGS (N=130,862) and 11 of 18 detected phyla.

#### sMGS enable high resolution metagenomics to track strains within hosts

To understand the precision of our sMGS method and the limits of reliably detecting persistent sMGS, we quantified the expected errors in our metagenomic reconstructed genomes. This was done on a real metagenomic test set, taking advantage of the thousands of longitudinally sampled gut metagenomes. We treated samples from the same host as biological replicates, as persistent strains are expected to remain genetically almost identical over the time frames sampled here (Duchêne et al., 2016). Longitudinal (and host family) samples harbouring the same strain of a given MGS were identified using monophyletic groups within the sMGS’ phylogeny (Figure 1C). This approach enabled us to classify sMGS into strains based on monophyly, which circumvents the necessity to find a clustering cut-off, such as average nucleotide identity (ANI). However, in practice this approach performs similarly to using stringent cut-offs (see STAR Methods). In our data, we identified 89,523 monophyletic sMGS groups, 26,047 occurring in 2 or more metagenomes. The genetic similarity among strains occurring in multiple samples was used to calculate an ANI of the same strains in different time points or family members.

We find that 95%, 74%, 59% and 55% of our 26,047 strains would still be detected at 99.9%, 99.99%, 99.999% and 100% ANI cut-offs. The median ANI of our detected strains was 100% (IQR 0.01), the average ANI 99.98% (± 0.12, Figure 1D). To put this into context, a recent benchmark of state-of-the-art metagenomic strain tools showed that the rate of detecting shared strains between fecal metagenomes of new-born twins, drops significantly below an ANI cut-off between 99.9% to 99.999% ANI, depending on tool being used (Olm et al., 2021). Bacterial mutation rates are in the range of 1e-8 to 1e-5 /nucleotide/year (Duchêne et al., 2016) and would fall within our achieved error tolerance.

For brevity, we will refer to MGS by species, and sMGS by strains in the main text.

#### Measures of persistence, family association and phylogeography

Within the human gut, bacterial persistence is the association of a bacterial strain with the same host over longer times. Here we define `strain persistence` as the longest time that a given strain was continuously observed across consecutive samples of the same host, divided by the time between the first and last observation of this host. In addition, we implemented a `strain resilience` and `annual persistence` measure, corresponding respectively to i) the fraction of consecutive samples of the same host, within which the same strain was detected, and ii) a survival probability of a strain surviving for a year, implemented via Kaplan-Meier survival statistics (Tables 1 and S1). Strain resilience was inversely related to the number of strains of a species detected in the same host (R=-0.97), thus being an approximation for strain replacement rates.

We calculated the `family association` of gut bacteria on 291 family pairs of host individuals. It should be noted that typical adult and infant microbiomes differ in abundance of bacteria (Ferretti et al., 2018; Korpela et al., 2018), which limited our ability to get ≥ 2X genome coverage for potentially shared strains. To address this potential bias, the family association score was calculated for each species as the average proportion of shared strains among host pairs that carried the same species at sufficient abundance (random timepoint). Note that the direction of strain transmission is not resolved in this analysis.

To calculate phylogeographic patters in the intraspecific strain phylogeny, two complementary approaches were used, by restricting analysis to one metagenome per family/host to avoid autocorrelations. `Geographic association` was calculated by correlating phylogenetic and geographic distance using a Mantel test. `Country association` tested if intraspecific phylogenetic distance were shorter within countries, than those between countries, using a perMANOVA test (detailed below).

#### Metagenomic assembly

All internal + external metagenomic samples (N=5,278) were assembled. All shotgun metagenomic reads were quality-filtered by removing reads shorter than 70% of the maximum expected read length (100 bp, 250 bp for miSeq data), an estimated accumulated error >2.5 with a probability of ≥0.01(Puente-Sanchez et al., 2015) or with an observed accumulated error >2, or >1 ambiguous position to assist assembly. If base quality dropped below 20 in a window of 15 bases at the 3′ end, or if the accumulated error exceeded 2, reads were trimmed. All these filter steps are integrated in sdm (Hildebrand et al., 2014). Human reads were removed from the metagenomic data, classifying raw reads with Kraken (Wood and Salzberg, 2014) against a custom database built on the human reference genome. Unclassified reads were further used in downstream analysis. In total 2.17e11 reads were filtered to 2.04e11 reads, used in subsequent analysis. sdm filtered paired reads were assembled using MegaHit (Li et al., 2015) with the parameters “--k-list 25,43,67,87,101,127”. The samples were assembled per individual (“co-assembly”) to find a balance between decreasing chimeric contigs, and increasing the chance of assembling low abundant genomes. The assembled scaffolds had an average N50 of 9,560 bp and a total size of 1e12 bp. Using Prodigal (Hyatt et al., 2010) in metagenomic mode, 1.4e9 genes were predicted on the contigs, of which half (6e8) were complete.

#### Abundance estimates of contigs and genes

To estimate the abundance of contigs, and subsequently genes, in each sample, unfiltered reads from a sample were mapped against the assembly of these reads (or co-assemblies of samples from the same host). Both low and good quality reads were mapped against the co-assemblies to estimate the abundances, since low quality filters will still accurately map to the assemblies in spite of several base errors. Bowtie2 v 2.3.4.1 (Langmead and Salzberg, 2012) was used for mapping, using the options “--no-unal --end-to-end --score-min L,-0.6,-0.6”. The resulting bam files were sorted, and duplicates removed and indexed using Samtools 1.3.1 (Li et al., 2009). Further, reads mapping with a mapping quality < 20, <95% nucleotide identity or <75% overall alignment length were filtered using custom Perl scripts. From these depth profiles were created using bedtools v2.21.0 (Quinlan and Hall, 2010) which were translated with a custom C++ program “rdCov” (https://github.com/hildebra/rdCover) into average coverage in a 50 bp window, per contig or per gene predicted on each contig, that were used in the gene catalog.

Further, “jgi_summarize_bam_contig_depths” from the MetaBAT2 package was used to translate bam files to abundances (per sample group), to be used in later steps for MetaBAT2 Binning (Kang et al., 2019).

#### Gene catalogue

Genes from assembled metagenomes were collated and separated into complete and incomplete genes, based on Prodigal v2.6.1 reporting (Hyatt et al., 2010). In a first step, the complete genes were clustered at 95% nucleotide identity, a commonly used cutoff in constructing gene catalogues (Sunagawa et al., 2015). For sequence clustering CD-HIT v4.6.1 (Fu et al., 2012) was used in est mode, employing parameters adapted to full-length genes: “-n 9 -G 1 -aS 0.95 -aL 0.6 -d 0 -c 0.95 -g 0”. This resulted in 14,083,686 clustered full-length genes, onto which the incomplete genes were mapped with Bowtie2 (Langmead and Salzberg, 2012). Incomplete genes mapping with at least 95% nucleotide identity were directly integrated into the initial clustering of complete genes. The remaining sequences were clustered as before with CD-HIT, but changing alignment length parameters to “-aL 0.3 -aS 0.8” to account for incomplete genes. Additionally, genes belonging to the 40 conserved marker genes were clustered separately, using clustering identity thresholds as described in (Mende et al., 2013). Merging marker gene clusters, incomplete, and complete clustered genes resulted in the gene catalogue, with a total of 23,137,742 non-redundant genes at 95% nucleotide identity cut-off. A gene abundance matrix was calculated using rtk (Saary et al., 2017).

The abundance of genes in each sample was calculated by backtracking clustered genes to their originating sample, and using the already computed gene abundances (see “Abundance estimates of contigs and genes“), thereby avoiding inflating the mapping space when mapping reads to a complete gene catalog. The gene abundance matrix was “decluttered” to remove redundant genes that were possibly originating from the same orthologue, but incorrectly clustered at nucleotide level. For this we first collated a list of “similar” genes, clustering the gene catalog at 90% AA identity with MMseqs2 (Steinegger and Söding, 2017). All thus clustered proteins were tested if they showed a co-excluding occurrence pattern (fisher’s exact test, p<1e-5), tested on gene pairs whose co-occurrence was in <10% of samples were either was observed. In total, 5.1e6 of 2.3e8 genes in the gene catalog were merged, based on their occurrence pattern. All genes in the gene catalog were functionally annotated to the eggNOG database (Powell et al., 2014) using Diamond (Buchfink et al., 2015) in blastp mode using options “-k 5 -e 1e-5 –sensitive”.

#### Binning

A combined binning approach was employed that uses single assembly binning (MAGs - metagenomic assembled genomes), gene catalogue binnings (Canopy clusters) and hierarchical clustering of candidate genes (hcl-clusters). The final set of genomes dereplicated at species level was the representative “MGS” (metagenomic species), used in all analysis unless otherwise mentioned.

To bin MAGs from single assemblies, MetaBAT2 v2.15 (Kang et al., 2019) was run on every metagenomic co-assembly with default parameters, except for the changed option “-min_contig_length 400”, using pre-computed contig abundances. These were quality-filtered using CheckM v1.0.11 (Parks et al., 2015). Based on MetaBAT2 bin predictions from each individual’s metagenome assembly, we discovered 22,091 bins at >80% completeness and <5% contamination rate. To pre-cluster MAGs likely representing the same species, the overlap in shared genes (gene catalogue genes) among MAGs was computed. MAGs were merged, if at least 30% or 300 of their genes overlapped. Merging was completed on MAGs of quality tiers to ensure that lower quality MAGs were merged into higher quality MAGs. We defined 4 quality tiers defined by completeness greater than 95, 90, 80, 60% and contamination less than 5, 5, 5, 10% for each of the 4 tiers, based on checkM scores, resulting in 1,002 dereplicated MAGs.

In parallel, the gene catalogue was clustered based on the co-occurrence of genes between samples, were using the canopy clustering approach (Nielsen et al., 2014). To avoid false positive clusters based on highly similar samples, such as would be expected in longitudinal samples from the same host individual, the gene abundance matrix was prefiltered to remove samples that had >0.15 Spearman correlation. The C++ program was run on the filtered and scaled gene abundance matrix, with parameters “--profile_measure 75Q -b --stop_criteria 100000 --filter_max_top3_sample_contribution 0.7 --max_canopy_dist 0.1 --max_merge_dist 0.1”. These canopy clusters were merged to dereplicated MAGs at the previously used 30% gene overlap and a minimum of 300 overlapping genes, but only creating an independent species cluster if >= 90% of its genes were independent (not occurring in dereplicated MAGs).

These dereplicated bins were further refined to “MGS”: using gene correlations and hierarchical clustering (Hildebrand et al., 2019). Briefly, for each dereplicated bin a set of core genes was extracted (occurring in >= 10% of all associated MAGs, or all genes of a canopy cluster) that were used to “fish” additional co-occurring genes from the gene catalogue. Genes correlating to these core genes at >0.75 Pearson correlation and >0.85 spearman rho were included in a set of putative genes, including now merged MetaBAT2 bins, canopy bins and genes correlating to their abundance at relaxed correlation values. These putative gene bins were clustered in R using a hierarchical clustering approach. Since in cases of low occurrence rates Spearman correlations gave better results, we implemented an automatic algorithm that used either Pearson or Spearman correlations for the hierarchical clustering step. From the hierarchical clustering of genes a sub-cluster was extracted that contained as many of 40 single-copy marker genes (Mende et al., 2013) as possible, selecting preferentially sub-clusters that had no duplicate copies of these genes present. This algorithm is implemented in the script “ClusterBinAbund.R”, available in MATAFILER. This final refinement step of dereplicated bins resulted in the final set of MGS (N=1,683), each bin representing a species as based on non-overlapping gene sets clustered at 95% identity (from the gene catalogue). These MGS were then again tested for completeness and contamination using checkM (Parks et al., 2015), filtering for 1,144 high quality MGS with >80% completeness and <5% contamination.

This process significantly improved the quality of the initially binned and dereplicated MAGs. The total number of high-quality (completeness >80%, contamination < 5%) MGS increased from 1,002 (MetaBAT2 only) to 1144 (MetaBAT2+canopy+refinement). Also, within these sets the quality was higher, as median contamination dropped from 1.07 to 0.67 and median completeness increased from 91.9 to 93.2 on 1002 and 1144 MGS, respectively. On median, 37/40 marker genes were found in the MGS genomes. Further, on median final MGS contained 131 more genes than the original dereplicated MAGs. Applying the quality criteria for high, medium, and low quality reconstructed genomes proposed by (Bowers et al., 2017), would result in 805 high quality, 665 mid quality and 176 low quality MGS (bearing in mind that no rRNA genes are included in gene catalogues and MGS gene clusters).

Using our de-novo assembly approach, we were able to obtain 1,144 high quality MGSs (>80% completeness, <5% contamination) that we included in the subsequent analyses. All MGSs could be assigned to known and candidate phyla (Figure 1B). Only ten archaeal MGS were found and all were from the orders Methanomassiliicoccales or Methanobacteriales. The vast majority of 1,034 bacterial MGS were Firmicutes (67% of all MGS, 52% assigned to Firmicutes_A as it is defined in GTDB taxonomy (Parks et al., 2018)), followed by Bacteroidota (17%), Proteobacteria (5.6%) and Actinobacteriota (4.4%); altogether 18 prokaryotic phyla were detected. We also discovered several new clades not yet represented in NCBI or GTDB, including a candidate bacterial class in the phylum Lentisphaerae that was represented by four MGS, 24 MGS without a family assignment and 196 MGS without a genus assignment.

#### MGS taxonomy and between MGS phylogenetic trees

Phylogenies of MGS were de novo calculated based on the amino acid (AA) sequences of 40 marker genes (Mende et al., 2013) extracted from each MGS (or less if not all 40 marker genes were present). These were aligned using MAFFT (Katoh and Standley, 2013) with default options, The multiple sequence alignment (MSA) was trimmed and backtranslated to nucleotides using Trimal (Capella-Gutiérrez et al., 2009) (options “-keepheader -ignorestopcodon -gt 0.1 -cons 60”). From the backtranslated, concatenated AA MSA, a phylogeny was reconstructed using IQ-TREE 1.6.3.a (Nguyen et al., 2015), with the options “-m GTR+F+I+G4 -B 1000”. The phylogeny was visualized using iTOL (Letunic and Bork, 2016).

MGS were taxonomically assigned using GTDB-TK and following the GTDB phylogeny (Parks et al., 2018). In addition the single copy marker genes from each MGS were matched to proGenomes (Mende et al., 2017), while all MGS genes were taxonomically annotated using kraken2 (Wood et al., 2019) (implemented in the script “taxPerMGS.pl” in MATAFILER, detailed in section “MGS abundance in metagenomes”). The GTDB taxonomic assignment was primarily used, unless only proGenomes or Kraken2 species level assignments were available, matching to the GTDB genus or family level assignment. This taxonomy was imposed on a phylogeny based on 40 conserved marker genes, allowing for a least-common-ancestor approach to obtain taxonomic assignments of unplaced taxa, at higher taxonomic levels. Using this approach, we still find several unclassified genera, families, and orders, but all MGS are assigned at least at phylum level.

#### Estimating MGS abundance in metagenomes

Taxonomic abundance within samples was estimated from the mean abundance of 40 universally present, single copy marker genes (MGs), found either in MGS or in reference genomes. This was derived from MGs clustered in the gene catalogue.

In detail, we assume that not all species present in the gut microbiome can be binned at a high quality, and wanted to use reference-based information to still estimate the abundance of these species in our samples, in order to obtain a more realistic abundance of microbial species. For this, we used the 40 single copy ubiquitously present marker genes (MG) that can be predicted using specI (Mende et al., 2013) in the gene catalogue, employing hidden markov models trained on broad taxonomic groups to detect MGs. This information was used to combine marker genes that were present in previously binned MGS, and those marker genes that could not be binned into MGS, to have an estimate of MGS, and non-MGS species present in each sample.

From this set of marker genes (some binned in MGS, others not), we used a similarity-based approach to identify known species within these, mapping all predicted genes with Lambda 1.9.3 (Hauswedell et al., 2014) onto MGs clustered to specI’s in the proGenomes database (Mende et al., 2017) and using a MG specific similarity cutoff (Mende et al., 2013). Hits were immediately accepted, if a metagenomic MG was hitting a single proGenomes specI at the required similarity threshold. However, since several metagenomic MGs had valid hits to multiple proGenomes MG’s, we supplemented the identification of a species using a coabundance approach, in a concept similar to canopy clustering (Nielsen et al., 2014). Briefly, for “complete” specI’s (>30 MG present or based on binned MGS annotations) we calculated the average profile across all MGs. The remaining MGs that were not uniquely assigned to a single specI (with >30 MG’s, or binned MGS), were correlated to existing specI profiles. Correlated MG’s were then tested for taxonomic similarity (being assigned to the same species or the same genus), and were combined to a new cluster if the pearson correlation coefficient was >0.9.

Additionally, at different phases in the clustering algorithm, within each specI, MGs were checked to correlate with the average profile (Spearman) < 0.9, or were removed as false positive assignments and iteratively tried to be added to different specI’s, as described above. This is implemented in the script “annotateMGwSpecIs.pl” available in MATAFILER. For analysis, mean abundance refers to non-zero mean abundance, excluding samples that do not show presence of a given species, unless otherwise mentioned. This was chosen to reflect a population of a given bacterium better, that the species usually reaches in normal ecosystems, excluding the prevalence of a species observed in our sample set. Prevalence was calculated as samples with >1% abundance of a given species

#### Characterisation of sporulation-related and oxygen-resistance genes in MGS

Core genes of MGS were annotated with EggNOG-mapper v2.0.0 based on eggNOG orthology data (Huerta-Cepas et al., 2019). Sequence searches were performed using Diamond v0.9.24.125 (Buchfink et al., 2015). KO annotations were retrieved from Eggnog-mapper annotations for each MGS. KOs related to spores or to oxygen resistance (catalases, peroxidases, super-oxide dismutases according to (Browne et al., 2017; Miller and Britigan, 1997; Rolfe et al., 1978)) were identified. The occurrences of all of these KOs were counted by MGS.

#### Strain-level metagenomics—within MGS phylogenies

To obtain strain-resolved MGS, we used conserved marker genes within a MGS, to construct a within-MGS phylogeny. This phylogeny was based on SNP-resolved gene orthologues (derived from the gene catalogue) from each sample where these genes were present at enough coverage. This approach is fundamentally different from aligning reads to reference genomes/genes, as it relies on assembled genes and multiple sequence alignments (MSA’s) instead of aligned reads, to describe genetic diversity. We chose this approach to a) avoid inflating the mapping space and b) reduce reference biases - to mitigate SNP calling errors typically introduced through mappings reads to a low similarity reference (Bush et al., 2019). The procedure from 1) SNP calling 2) conspecific strain detection 3) MGS gene selection 4) multiple sequence alignment and phylogeny is described in the following.

##### Determining nucleotide variants per sample

Reads were mapped against the complete metagenomic co-assembly of a given sample group. From the filtered bam files (see above), nucleotide variants per biological sample were computed using Bcftools mpileup 1.9 (Li, 2011) with the following additional options “--count-orphans --min-BQ 30 -d 12000 --skip-indels --min-MQ 20 -a DP,AD,ADF,ADR,SP “. The output vcf file was processed using a custom Perl script (Hildebrand et al., 2019), to calculate the consensus fasta sequence for each assembled contig in a given sample. The minimum coverage was set to 2 reads per position. A 0.501 consensus threshold was used, to avoid introducing a reference bias in the consensus sequence and to filter reads assignments at a depth of 2, that have a 50% allele frequency. Further, using the 50% allele frequency cut-off addresses conspecific strains present in the same host, by only estimating the genome of the most abundant strain. If a second strain would in another time point arise to become the dominant strain, this would be immediately inferable from the within-host diversity and flagged as a strain exchange. However, to avoid conspecific strains being present in the same time point at comparable abundances, which would introduce noise into our calculations, we specifically filtered all contigs in a sample, that might originate from conspecific strains.

#### Detecting conspecific strains

To filter out sequences that likely arose from >1 strain of the same species present in the same sample, we tested in each contig the frequency of non-reference alleles. Since fixed SNPs (>0.80 or <0.20 allele frequency) are simply a function of differences between reference sequence and strains present in a sample, these alleles were completely ignored. Instead, we only focused on mid ranging frequencies (0.20 to 0.80), in analogy to a strain delineation threshold of 1% base differences, we approximated the probability of conspecific strains (P_c_) in a sample corresponding to the % of mid-frequency positions, out of covered positions. The likelihood of a specific gene being confounded by conspecific strain signature was conservatively calculated as:

P_gc_ = P_c_^∗^ N_midF_^∗^ 100 / L_g_

where N_midF_ is the number of SNPs at midrange frequencies and L_g_ the gene length of a given gene.

#### MGS representative genes and outgroups

For each MGS of sufficient quality, initially 300 genes were selected, that were either part of the conserved 40 MG’s (Mende et al., 2013), or were ubiquitiously present (a sorted list of gene presence was used, to select the top 300 genes, minus the number of present MG genes). To avoid conspecific strains, two checks were performed: 1) a gene catalogue gene was completely excluded from further analysis, if it was represented by >1 gene within a sample in more than 5% of cases 2) a gene within a sample was excluded, if P_gc_ > 0.1.

An outgroup for each MGS was automatically chosen based on the between MGS phylogeny: the closest neighbour that was further than 0.1 evolutionary distance from the current MGS was automatically extracted from this phylogeny, using custom R scripts. To obtain corresponding genes between in- and outgroup, we used the functional assignments of the gene catalogue: in the outgroup we obtained those genes that were either of the same MG assignment, or (for non-MG genes) of the same eggNOG assignment.

##### Alignment and phylogeny

From ingroup and outgroup genes, multiple sequence alignments (MSA’s) per gene category were created (independently of each other), using AA sequences and mafft (Katoh and Standley, 2013) with default parameters. MSA’s suffer from their own technical idiosyncrasies, which we tried to mitigate through extensive automatic filtering. First, MSA’s were back-translated and filtered using trimal (Capella-Gutiérrez et al., 2009), with the parameters “-ignorestopcodon -gt 0.1 -cons 60”. Second, we observed sporadically occurring “frameshifts” in few MSA’s: whole sections of a gene were shifted by 3 nucleotides (typically the 3′ or 5′ part), leading to very low sequence identities on part of the MSA. To correct for these, a custom C++ program was implemented (available on https://github.com/hildebra/MSAfix). This calculates iteratively for each sequence the identity to all other sequences in a 150bp window; if such a window falls below the expected identity threshold (E[id]) for at least 50 nucleotides, that region of the gene is masked. If more than 60% of nucleotides were masked, the entire sequence was discarded. The expected identity threshold was calculated as

E[id] = (IDi) - ((1 - IDi)^∗^0.25) - max(0.1,σ^∗^3)

With IDi being the average %identity of sequence i to all other sequences in the MSA, σ the standard deviation of %identities among sequences. After these corrections, the final set of genes to be used in the phylogeny was selected, choosing those genes that were at least 400 bp long, were at least 75% non-N characters. A strain (that is a sample representing a MGS) was excluded from further analysis, if not at least 10% of overall nucleotides were covered (non-N) or not at least 20% of genes used in the phylogeny were present.

From these filtered MSA’s, a partitioned phylogeny was created using IQ-tree (Nguyen et al., 2015) in fast mode, to handle the computational complexity of this dataset (with some trees containing >4,000 tips), with the options “-m GTR+F+I+G4 -seed 678 -alrt 1000 -fast”.

To capture statistics on the tree structure imbalances, we measured Sackins index. To measure these on our rooted trees, we used the R-package “apTreeshape”, using pda normalization to account for tree size.

#### Strain individual, family, and geographic stability

All samples related to fecal microbiota transfers were excluded for host/family/geographic association statistics. For each species we calculated the fraction of stable phylogenetic clustering of strains detected in a given individual, defined as either the same sMGS (strain) between two samples, or different sMGS, where the MGS (species) was sufficiently abundant to allow for strain identification. We developed three separate approaches to measure if a strain was in the same cluster using: first we set a hard cutoff at 0.01 evolutionary distance, based on our nucleotide derived phylogenetic trees. Second, we used the tree specific cutoff at 10% quantile of all distances in a species’ tree (usually considerably lower than 0.01, see Figure 1D for median values). Third, we used a phylogeny-based approach, where we only confirmed that the closest relative to a given leaf (sMGS) was of the same individual. From this latter approach the within-individual evolutionary distance was derived at 95% quantile, which was subsequently used to cluster remaining leaf’s if they fall within this distance to their closest strain from the same individual. The second and third approach were virtually identical (Rho=0.99), but the hard cutoff at 0.01 led to divergent results (Rho~0.77 to either). The third approach was used for the majority of analysis, unless otherwise mentioned.

To estimate strain resilience, sMGS assignments between all consecutive timepoints within the same individual host were compared. Strain resilience is the number of consecutive timepoint with the same sMGS (Ncts) divided by the number of consecutive timepoints with a different sMGS (Nctd); timepoints were the MGS could not be detected were excluded. Ncts and Nctd were summed across individuals, divided by each other to obtain a fraction and multiplied by 100 to obtain a percentage strain resilience per MGS. Strain persistence was calculated similarly, but instead of Ncts and Nctd representing the number of cases of similar/dissimilar sMGS, these represented the accumulated days between two time points, Dcts and Dctd. While it was straightforward to calculate Dcts as consecutive days with the same sMGS (Dcts += T_x+1_ – T_x_, T being relative timepoint in individual, x the considered sample), Dctd could represent a strain exchange happening on any time point between last and current observation. Therefore, we used a maximum likelihood approach, assuming the strain exchange happening in the middle between timepoints (Dcts += T_x+1_ – T_x_/2 and Dctd += T_x+1_ – T_x_/2). Annual survival rate was estimated using survival statistics. Here the R-package “survival” and function “Surv” was used on the longest timeframe within each individual, where we could detect a strain (right censoring). To estimate the percentage of strains that survived for 365 (or other times) days, we used the stepfun and survest R functions.

Family persistence was estimated from the same within-MGS phylogenies, using the same algorithm described above. This was used to obtain strains shared between individuals of the same family (any timepoint was valid for this). If the family members were both adults, this was defined horizontal, if an adult and infant/teenager shared a strain, this was defined as vertical strain sharing.

Geographic stability was tested twofold, but in either case we reduced the sample set to include only one sample per individual, or per family if other family members were within out dataset. This representative sample was chosen randomly, as long as it contained the MGS, even if we detected >1 strain per sample, to avoid any false positive signal due to incomplete strain delineation. We tested the association to a specific country, by testing if within-country genetic distance was significantly smaller than between-country distance, using a perMANOVA, as implemented in the vegan R package (Oksanen et al., 2014). We tested the geographic association by testing for a significant correlation between geographic distance and genetic distance, using a mantel test. As genetic distance we used the cophenetic distance between leaves of the within-MGS phylogeny. As all metagenomic samples were from anonymised individuals, we inferred GPS coordinates for either the sampling hospital (if detailed in original publications) or the country the study was conducted in. For multi-country studies, the information of participant origin was obtained from published metadata or directly from the authors. Geographic distance was estimated from GPS coordinates of the samples origin, using the distm function from the geosphere R-package with option “fun = distHaversine”.

#### Estimating population genetic parameters of MGS in metagenomic samples

All population genetic parameters (Tajima’s D, Θ, π, d_N_/d_S_, p_N_/p_S_) were calculated from the same set of core genes used for strain delineation (see above). This set was optimally aligned to our aim of describing the evolutionary forces underlying the stably transferred core genome, experienced by the species over longer time scales. This set also has the advantage of being less likely subject to horizontal gene transfer (HGTs) and being present in multiple, paralogous copies. Based on MSA’s described above, all population genetic parameters were calculated separately for each gene, after filtering for genes present in too few samples and conspecific/paralogous gene filtering. This resulted in 64 genes on median per MGS, and the median value for each population genetic parameters was used to represent said parameter for each MGS (see Table S4 for detailed listing).

Estimates were inferred either directly from the MSAs (Tajima’s D, Θ, π, d_N_/d_S_) or MSAs plus derived phylogeny to estimate p_N_/p_S_ (Murrell et al., 2013). Tajima’s D, Θ, π and p_N_/p_S_ were calculated solely from nucleotide multiple sequence alignments within a MGS, while for d_N_/d_S_ sequences from an outgroup MGS were included. However, within-MGS sequences were later downsampled to account for potential biases (see further). Tajima’s D, Θ, π were calculated using the R packages PEGAS and APE (Paradis, 2010; Paradis et al., 2004), using the functions “tajima.test”, ”theta.s” and “nuc.div”.

Tajima’s D, Θ, π at synonymous and non-synonymous sites (Tajima’s D_S_, Θ_S_, π_S_, Tajima’s D_N_, Θ_N_, π_N_ ) were calculated based only on sites of the MSA, for simplicity represented by either 4-fold (synonymous) or 0-fold (non-synonymous) degenerate coding DNA positions, as determined with subfunctions from “dnds” (R-package APE). To estimate the strength of purifying selection acting on of polymorphisms potentially segregating within the bacterial species (p_N_/p_S_), we chose to use a Bayesian approach to estimate p_N_/p_S_, using a maximum likelihood approach implemented in FUBAR (Murrell et al., 2013). After consultation with one of the software’s author (SK Pond), we used grid-estimated p_N_ and p_S_ values, corrected by their posterior probability.

We found that some of our estimates correlated strongly to the number of samples included per MGS (Figure S4). While this could be a genuine biological signal it may be possible that the number of sequences in an analysis might bias results for technical reasons. To address this, we corrected for differences in sampling depth (i.e. number of genomes available per MGS) and autocorrelation in time series data, by 1) randomly selecting 1 sample per host-individual and 2) randomly selecting from this set n=10,20,30,100,200 or 500 genes. Within each of these randomly downsampled (and therefore normalized) subsets the correlation between p_N_/p_S_ as well as Θ (and other tested variables, data not shown) to the number of samples remained stable, indicating that a biological signal was present (data not shown). We choose to use n=20 in the remaining analysis, a downsampling that still represented 452 MGS, mostly overlapping with the 441 MGS chosen to investigate persistence, where key population genetic estimates were stable at different downsamplings.

To estimate d_N_/d_S_, that is the ratio of between species nucleotide divergence at non-synonymous and synonymous sites, a nucleotide sequences of an outgroup species was included. For this we reused the outgroup included to build the within-MGS phylogeny (see section above) to obtain and align orthologous genes of this outgroup MGS. Based on the MSA used to delineate strains. d_N_/d_S_ was estimated using the function “dnds” from APE (Paradis et al., 2004) in R, by sequentially comparing the outgroup to each ingroup sequence to obtain estimates of d_N_/d_S_, d_N_, d_S_. The median of these across all ingroup sequences was taken to represent the gene’s d_N_/d_S_, d_N_, d_S_ values for a specific MGS.

### Quantification and statistical analysis

All statistical analysis and plotting of graphs was conducted in R 3.6.2, unless otherwise mentioned. All correlations and tests of correlations used spearman correlations, unless otherwise mentioned, using function “cor.test” in base R and all reported correlations are spearman Rho values. All tests comparing groups used a two-sided Wilcoxon rank-sum test, or a Kruskal-wallis test in case of more than three categories, using functions “wilcox.test” and “kruskal.test” implemented in base R. Whenever multiple tests were conducted, these were multiple testing corrected, using Benjamin-Hochberg multiple testing correction with the base R function “p.adjust”. Partial correlations were calculated using function pcor.test from package “ppcor” ver 1.1. Correlelograms were calculated with function “corrgram” package “corrgram” ver 1.13, using argument cor.method="spearman". Enrichments of persistence or resilience of taxa in hosts of differing age, delivery mode or antibiotic treatment, as well as taxa enriched in vertical compared to horizontal strain sharing were calculated based on count data (observed cases with the same / different strain). For this a Fisher’s exact test was used, as implemented in the R function “fisher.test”. Strain persistence is directly calculated from observations between consecutive time points, that we interpreted as independent observations that could be used directly in Fisher’s exact test. However, strain persistence is based on days a strain is consecutively observed. Therefore, we used the information of total observations in strain resilience, to downscale consecutive time to reflect the total N obtain from strain resilience. P-values were not multiple testing corrected, as we rather sought an overview of plausible enrichments or depletions of taxa upon antibiotic exposure.

“Antibiotic Impact” on persistence/resilience was determined by comparing the persistence or resilience of a species in hosts that were not exposed to antibiotics to these values in hosts exposed to antibiotics. The ratio of the averages was taken as “impact” that antibiotics will have on the persistence/resilience.

Strain persistence and resilience in age windows was calculated by first calculating either statistic for a patient and then calculating the median age of the individual. Median age and persistence/resilience were averaged in age windows to understand the changes in persistence and resilience that come with host age, for the different microbial species in our data.

### Additional resources

Gene catalog and sample metadata are available on http://vm-lux.embl.de/∼hildebra/Drama_GC.

## Supplemental information

Document S1. Figures S1–S4

Table S1. Phylum and genera persistence and associations, related to Figure 2 The main association characteristics for 440 MGS are summarized at phylum and genus level. “Geographic association^∗^” = correlation is only shown for significant MGS.

Table S2. Strain persistence is affected by antibiotics and family sharing, related to Figure 2 (A) Fisher’s exact test was used to test at different taxonomic levels (domain phylum, class, order, family, genus, species) if the p value of the OR between Same/Different strains was different between antibiotic and non-antibiotic treated hosts. Only for taxa with a p value < 0.05 this odds-ratio is reported. (B) Vertical (parent-child) transmission of strains is often enriched compared to horizontal (parent-parent) transmission. This was tested using a Fisher’s exact test (p value) followed by Benjamini-Hochberg multiple testing corrected. (C) Host and Family association characteristics are listed for all 440 MGS, including their taxonomic assignment. “Ncompar,” the number of witin-family pairs of hosts, where an MGS could be analysed in a family context (horizontal/vertical transmission rate, family association).

Table S3. Cohorts used in meta-analysis, related to Figure 1 (A) Studies used in meta-analysis. Accession codes, number of samples, number of individuals, mean and standard deviation of age, and notes to each cohort are listed. (B) Metadata for meta-analysis cohort, including individual, time point in days relative to first sampling, family, sex, country of host and the total read pairs (before processing), age, where available for each sample. Negative age values indicate preterm infants. Further we indicated samples from the new cohort that were previously published.

Table S4. Population genetic parameters for gut bacteria, related to Figure 3 These are estimated for each MGS. nuclDiv = π, dnds = d_N_/d_S_, pnps = p_N_/p_S_, N, non-synonymous sites; S, synonymous sites.

Document S2. Article plus supplemental information

## Footnotes

Supplemental information can be found online at https://doi.org/10.1016/j.chom.2021.05.008.

## References

[bib1] Asnicar F., Manara S., Zolfo M., Truong D.T., Scholz M., Armanini F., Ferretti P., Gorfer V., Pedrotti A., Tett A., Segata N. (2017). Studying vertical microbiome transmission from mothers to infants by strain-level metagenomic profiling. mSystems.

[bib2] Baas-Becking L.G.M. (1934). Geobiology of Inleiding Tot de Milieukunde.

[bib3] Bäckhed F., Roswall J., Peng Y., Feng Q., Jia H., Kovatcheva-Datchary P., Li Y., Xia Y., Xie H., Zhong H. (2015). Dynamics and stabilization of the human gut microbiome during the first year of life. Cell Host Microbe.

[bib4] Bengtsson-Palme J., Angelin M., Huss M., Kjellqvist S., Kristiansson E., Palmgren H., Larsson D.G., Johansson A. (2015). The human gut microbiome as a transporter of antibiotic resistance genes between continents. Antimicrob. Agents Chemother..

[bib5] Bowers R.M., Kyrpides N.C., Stepanauskas R., Harmon-Smith M., Doud D., Reddy T.B.K., Schulz F., Jarett J., Rivers A.R., Eloe-Fadrosh E.A. (2017). Minimum information about a single amplified genome (MISAG) and a metagenome-assembled genome (MIMAG) of bacteria and archaea. Nat. Biotechnol..

[bib6] Brito I.L., Gurry T., Zhao S., Huang K., Young S.K., Shea T.P., Naisilisili W., Jenkins A.P., Jupiter S.D., Gevers D., Alm E.J. (2019). Transmission of human-associated microbiota along family and social networks. Nat. Microbiol..

[bib7] Browne H.P., Neville B.A., Forster S.C., Lawley T.D. (2017). Transmission of the gut microbiota: spreading of health. Nat. Rev. Microbiol..

[bib8] Buchfink B., Xie C., Huson D.H. (2015). Fast and sensitive protein alignment using DIAMOND. Nat. Methods.

[bib9] Bush S.J., Foster D., Eyre D.W., Clark E.L., Maio N. De, Shaw L.P., Stoesser N., Peto T.E.A., Crook D.W., Walker A.S. (2019). Genomic diversity affects the accuracy of bacterial SNP calling pipelines. bioRxiv.

[bib10] Capella-Gutiérrez S., Silla-Martínez J.M., Gabaldón T. (2009). trimAl: a tool for automated alignment trimming in large-scale phylogenetic analyses. Bioinformatics.

[bib11] Charlesworth B. (2009). Fundamental concepts in genetics: effective population size and patterns of molecular evolution and variation. Nat. Rev. Genet..

[bib12] Chu D.M., Ma J., Prince A.L., Antony K.M., Seferovic M.D., Aagaard K.M. (2017). Maturation of the infant microbiome community structure and function across multiple body sites and in relation to mode of delivery. Nat. Med..

[bib13] Costea P.I., Coelho L.P., Sunagawa S., Munch R., Huerta-Cepas J., Forslund K., Hildebrand F., Kushugulova A., Zeller G., Bork P. (2017). Subspecies in the global human gut microbiome. Mol. Syst. Biol..

[bib14] Dearlove B.L., Frost S.D.W. (2015). Measuring asymmetry in time-stamped phylogenies. PLoS Comput. Biol..

[bib15] Dethlefsen L., McFall-Ngai M., Relman D.A. (2007). An ecological and evolutionary perspective on human-microbe mutualism and disease. Nature.

[bib16] Didelot X., Nell S., Yang I., Woltemate S., van der Merwe S., Suerbaum S. (2013). Genomic evolution and transmission of Helicobacter pylori in two South African families. Proc. Natl. Acad. Sci. USA.

[bib17] Duchêne S., Holt K.E., Weill F.X., Le Hello S., Hawkey J., Edwards D.J., Fourment M., Holmes E.C. (2016). Genome-scale rates of evolutionary change in bacteria. Microb. Genom..

[bib18] Faith J.J., Guruge J.L., Charbonneau M., Subramanian S., Seedorf H., Goodman A.L., Clemente J.C., Knight R., Heath A.C., Leibel R.L. (2013). The long-term stability of the human gut microbiota. Science.

[bib19] Ferretti P., Pasolli E., Tett A., Asnicar F., Gorfer V., Fedi S., Armanini F., Truong D.T., Manara S., Zolfo M. (2018). Mother-to-infant microbial transmission from different body sites shapes the developing infant gut microbiome. Cell Host Microbe.

[bib20] Foster K.R., Schluter J., Coyte K.Z., Rakoff-Nahoum S. (2017). The evolution of the host microbiome as an ecosystem on a leash. Nature.

[bib21] Fu L., Niu B., Zhu Z., Wu S., Li W. (2012). CD-HIT: accelerated for clustering the next-generation sequencing data. Bioinformatics.

[bib22] Garud N.R., Good B.H., Hallatschek O., Pollard K.S. (2019). Evolutionary dynamics of bacteria in the gut microbiome within and across hosts. PLoS Biol.

[bib23] Hauswedell H., Singer J., Reinert K. (2014). Lambda: the local aligner for massive biological data. Bioinformatics.

[bib24] Heintz-Buschart A., May P., Laczny C.C., Lebrun L.A., Bellora C., Krishna A., Wampach L., Schneider J.G., Hogan A., De Beaufort C., Wilmes P. (2016). Integrated multi-omics of the human gut microbiome in a case study of familial type 1 diabetes. Nat. Microbiol..

[bib25] Hildebrand F., Moitinho-Silva L., Blasche S., Jahn M.T., Gossmann T.I., Huerta-Cepas J., Hercog R., Luetge M., Bahram M., Pryszlak A. (2019). Antibiotics-induced monodominance of a novel gut bacterial order. Gut.

[bib26] Hildebrand F., Tadeo R., Voigt A.Y., Bork P., Raes J. (2014). LotuS: an efficient and user-friendly OTU processing pipeline. Microbiome.

[bib27] Huerta-Cepas J., Szklarczyk D., Heller D., Hernández-Plaza A., Forslund S.K., Cook H., Mende D.R., Letunic I., Rattei T., Jensen L.J. (2019). EggNOG 5.0: A hierarchical, functionally and phylogenetically annotated orthology resource based on 5090 organisms and 2502 viruses. Nucleic Acids Res.

[bib28] Human Microbiome Project Consortium (2012). Structure, function and diversity of the healthy human microbiome. Nature.

[bib29] Hyatt D., Chen G.L., Locascio P.F., Land M.L., Larimer F.W., Hauser L.J. (2010). Prodigal: prokaryotic gene recognition and translation initiation site identification. BMC Bioinformatics.

[bib30] Kang D.D., Li F., Kirton E., Thomas A., Egan R., An H., Wang Z. (2019). MetaBAT 2: an adaptive binning algorithm for robust and efficient genome reconstruction from metagenome assemblies. PeerJ.

[bib31] Kaoutari A. El, Armougom F., Gordon J.I., Raoult D., Henrissat B. (2013). The abundance and variety of carbohydrate-active enzymes in the human gut microbiota. Nat. Rev. Microbiol..

[bib32] Katoh K., Standley D.M. (2013). MAFFT multiple sequence alignment software version 7: improvements in performance and usability. Mol. Biol. Evol..

[bib33] Korpela K., Costea P., Coelho L.P., Kandels-Lewis S., Willemsen G., Boomsma D.I., Segata N., Bork P. (2018). Selective maternal seeding and environment shape the human gut microbiome. Genome Res.

[bib34] Kostic A.D., Gevers D., Siljander H., Vatanen T., Hyötyläinen T., Hämäläinen A.M., Peet A., Tillmann V., Pöhö P., Mattila I. (2015). The dynamics of the human infant gut microbiome in development and in progression toward type 1 diabetes. Cell Host Microbe.

[bib35] Kujawska M., La Rosa S.L., Roger L.C., Pope P.B., Hoyles L., McCartney A.L., Hall L.J. (2020). Succession of Bifidobacterium longum strains in response to a changing early life nutritional environment reveals dietary substrate adaptations. iScience.

[bib36] Kushugulova A., Forslund S.K., Costea P.I., Kozhakhmetov S., Khassenbekova Z., Urazova M., Nurgozhin T., Zhumadilov Z., Benberin V., Driessen M. (2018). Metagenomic analysis of gut microbial communities from a Central Asian population. BMJ Open.

[bib37] Langmead B., Salzberg S.L. (2012). Fast gapped-read alignment with Bowtie 2. Nat. Methods.

[bib38] Lee S.T.M., Kahn S.A., Delmont T.O., Shaiber A., Esen Ö.C., Hubert N.A., Morrison H.G., Antonopoulos D.A., Rubin D.T., Eren A.M. (2017). Tracking microbial colonization in fecal microbiota transplantation experiments via genome-resolved metagenomics. Microbiome.

[bib39] Letunic I., Bork P. (2016). Interactive tree of life (iTOL) v3: an online tool for the display and annotation of phylogenetic and other trees. Nucleic Acids Res.

[bib40] Li D., Liu C.M., Luo R., Sadakane K., Lam T.W. (2015). MEGAHIT: an ultra-fast single-node solution for large and complex metagenomics assembly via succinct de Bruijn graph. Bioinformatics.

[bib41] Li H. (2011). A statistical framework for SNP calling, mutation discovery, association mapping and population genetical parameter estimation from sequencing data. Bioinformatics.

[bib42] Li H., Handsaker B., Wysoker A., Fennell T., Ruan J., Homer N., Marth G., Abecasis G., Durbin R., 1000 Genome Project Data Processing Subgroup (2009). The Sequence Alignment/Map format and SAMtools. Bioinformatics.

[bib43] Li S.S., Zhu A., Benes V., Costea P.I., Hercog R., Hildebrand F., Huerta-Cepas J., Nieuwdorp M., Salojärvi J., Voigt A.Y. (2016). Durable coexistence of donor and recipient strains after fecal microbiota transplantation. Science.

[bib44] Mehta R.S., Abu-Ali G.S., Drew D.A., Lloyd-Price J., Subramanian A., Lochhead P., Joshi A.D., Ivey K.L., Khalili H., Brown G.T. (2018). Stability of the human faecal microbiome in a cohort of adult men. Nat. Microbiol..

[bib45] Mende D.R., Letunic I., Huerta-Cepas J., Li S.S., Forslund K., Sunagawa S., Bork P. (2017). proGenomes: a resource for consistent functional and taxonomic annotations of prokaryotic genomes. Nucleic Acids Res.

[bib46] Mende D.R., Sunagawa S., Zeller G., Bork P. (2013). Accurate and universal delineation of prokaryotic species. Nat. Methods.

[bib47] Miller R.A., Britigan B.E. (1997). Role of oxidants in microbial pathophysiology. Clin. Microbiol. Rev..

[bib48] Montano V., Didelot X., Foll M., Linz B., Reinhardt R., Suerbaum S., Moodley Y., Jensen J.D. (2015). Worldwide population structure, long-term demography, and local adaptation of Helicobacter pylori. Genetics.

[bib49] Moran N.A., Ochman H., Hammer T.J. (2019). Evolutionary and ecological consequences of gut microbial communities. Annu. Rev. Ecol. Evol. Syst..

[bib50] Murrell B., Moola S., Mabona A., Weighill T., Sheward D., Kosakovsky Pond S.L., Scheffler K. (2013). FUBAR: a fast, unconstrained Bayesian AppRoximation for inferring selection. Mol. Biol. Evol..

[bib51] Nayfach S., Rodriguez-Mueller B., Garud N., Pollard K.S. (2016). An integrated metagenomics pipeline for strain profiling reveals novel patterns of bacterial transmission and biogeography. Genome Res.

[bib52] Nguyen L.T., Schmidt H.A., von Haeseler A., Minh B.Q. (2015). IQ-TREE: a fast and effective stochastic algorithm for estimating maximum-likelihood phylogenies. Mol. Biol. Evol..

[bib53] Nielsen H.B., Almeida M., Juncker A.S., Rasmussen S., Li J., Sunagawa S., Plichta D.R., Gautier L., Pedersen A.G., Le Chatelier E. (2014). Identification and assembly of genomes and genetic elements in complex metagenomic samples without using reference genomes. Nat. Biotechnol..

[bib54] Oksanen J., Blanchet F.G., Kindt R., Legendre P., Minchin P.R., O'Hara R.B., Simpson G.L., Solymos P., Stevens M.H.H., Szoecs E., Wagner H. (2014). vegan: community ecology package. https://CRAN.R-project.org/package=vegan.

[bib55] Olm M.R., Crits-Christoph A., Bouma-Gregson K., Firek B.A., Morowitz M.J., Banfield J.F. (2021). inStrain profiles population microdiversity from metagenomic data and sensitively detects shared microbial strains. Nat. Biotechnol..

[bib56] Palleja A., Mikkelsen K.H., Forslund S.K., Kashani A., Allin K.H., Nielsen T., Hansen T.H., Liang S., Feng Q., Zhang C. (2018). Recovery of gut microbiota of healthy adults following antibiotic exposure. Nat. Microbiol..

[bib57] Paradis E. (2010). pegas: an R package for population genetics with an integrated-modular approach. Bioinformatics.

[bib58] Paradis E., Claude J., Strimmer K. (2004). APE: analyses of phylogenetics and evolution in R language. Bioinformatics.

[bib59] Parks D.H., Chuvochina M., Waite D.W., Rinke C., Skarshewski A., Chaumeil P.A., Hugenholtz P. (2018). A standardized bacterial taxonomy based on genome phylogeny substantially revises the tree of life. Nat. Biotechnol..

[bib60] Parks D.H., Imelfort M., Skennerton C.T., Hugenholtz P., Tyson G.W. (2015). CheckM: assessing the quality of microbial genomes recovered from isolates, single cells, and metagenomes. Genome Res.

[bib61] Podlesny D., Fricke W.F. (2021). Strain inheritance and neonatal gut microbiota development: A meta-analysis. Int. J. Med. Microbiol..

[bib62] Powell S., Forslund K., Szklarczyk D., Trachana K., Roth A., Huerta-Cepas J., Gabaldón T., Rattei T., Creevey C., Kuhn M. (2014). eggNOG v4.0: nested orthology inference across 3686 organisms. Nucleic Acids Res.

[bib63] Puente-Sanchez F., Aguirre J., Parro V., Puente s., F., Aguirre J. (2015). A novel conceptual approach to read-filtering in high-throughput amplicon sequencing studies. Nucleic Acids Res..

[bib64] Quinlan A.R., Hall I.M. (2010). BEDTools: a flexible suite of utilities for comparing genomic features. Bioinformatics.

[bib65] Ramiro R.S., Durão P., Bank C., Gordo I. (2020). Low mutational load and high mutation rate variation in gut commensal bacteria. PLoS Biol..

[bib66] Raymond F., Ouameur A.A., Déraspe M., Iqbal N., Gingras H., Dridi B., Leprohon P., Plante P.L., Giroux R., Bérubé È. (2016). The initial state of the human gut microbiome determines its reshaping by antibiotics. ISME J..

[bib67] Rolfe R.D., Hentges D.J., Campbell B.J., Barrett J.T. (1978). Factors related to the oxygen tolerance of anaerobic bacteria. Appl. Environ. Microbiol..

[bib68] Saary P., Forslund K., Bork P., Hildebrand F. (2017). RTK: efficient rarefaction analysis of large datasets. Bioinformatics.

[bib69] Schloissnig S., Arumugam M., Sunagawa S., Mitreva M., Tap J., Zhu A., Waller A., Mende D.R., Kultima J.R., Martin J. (2013). Genomic variation landscape of the human gut microbiome. Nature.

[bib70] Scholz M., Ward D.V., Pasolli E., Tolio T., Zolfo M., Asnicar F., Truong D.T., Tett A., Morrow A.L., Segata N. (2016). Strain-level microbial epidemiology and population genomics from shotgun metagenomics. Nat. Methods.

[bib71] Shao Y., Forster S.C., Tsaliki E., Vervier K., Strang A., Simpson N., Kumar N., Stares M.D., Rodger A., Brocklehurst P. (2019). Stunted microbiota and opportunistic pathogen colonization in caesarean-section birth. Nature.

[bib72] Smith J.M., Haigh J. (1974). The hitch-hiking effect of a favourable gene. Genet. Res..

[bib73] Steinegger M., Söding J. (2017). MMseqs2 enables sensitive protein sequence searching for the analysis of massive data sets. Nat. Biotechnol..

[bib74] Sunagawa S., Coelho L.P., Chaffron S., Kultima J.R., Labadie K., Salazar G., Djahanschiri B., Zeller G., Mende D.R., Alberti A. (2015). Ocean plankton. Structure and function of the global ocean microbiome. Science.

[bib75] Tajima F. (1989). Statistical method for testing the neutral mutation hypothesis by DNA polymorphism. Genetics.

[bib76] Truong D.T., Tett A., Pasolli E., Huttenhower C., Segata N. (2017). Microbial strain-level population structure and genetic diversity from metagenomes. Genome Res..

[bib77] Vatanen T., Kostic A.D., D’Hennezel E., Siljander H., Franzosa E.A., Yassour M., Kolde R., Vlamakis H., Arthur T.D., Hämäläinen A.-M. (2016). Variation in microbiome LPS immunogenicity contributes to autoimmunity in humans. Cell.

[bib78] Voigt A.Y., Costea P.I., Kultima J.R., Li S.S., Zeller G., Sunagawa S., Bork P. (2015). Temporal and technical variability of human gut metagenomes. Genome Biol..

[bib79] Ward D.V., Scholz M., Zolfo M., Taft D.H., Schibler K.R., Tett A., Segata N., Morrow A.L. (2016). Metagenomic sequencing with strain-level resolution implicates uropathogenic E. coli in necrotizing enterocolitis and mortality in preterm infants. Cell Rep..

[bib80] Willmann M., El-Hadidi M., Huson D.H., Schütz M., Weidenmaier C., Autenrieth I.B., Peter S. (2015). Antibiotic selection pressure determination through sequence-based metagenomics. Antimicrob. Agents Chemother..

[bib81] Wood D.E., Lu J., Langmead B. (2019). Improved metagenomic analysis with Kraken 2. Genome Biol..

[bib82] Wood D.E., Salzberg S.L. (2014). Kraken: ultrafast metagenomic sequence classification using exact alignments. Genome Biol..

[bib83] Xie H., Guo R., Zhong H., Feng Q., Lan Z., Qin B., Ward K.J., Jackson M.A., Xia Y., Chen X. (2016). Shotgun metagenomics of 250 adult twins reveals genetic and environmental impacts on the gut microbiome. Cell Syst..

[bib84] Yassour M., Jason E., Hogstrom L.J., Arthur T.D., Tripathi S., Siljander H., Selvenius J., Oikarinen S., Hyöty H., Virtanen S.M. (2018). Strain-level analysis of mother-to-child bacterial transmission during the first few months of life. Cell Host Microbe.

[bib85] Yassour M., Vatanen T., Siljander H., Hämäläinen A.M., Härkönen T., Ryhänen S.J., Franzosa E.A., Vlamakis H., Huttenhower C., Gevers D. (2016). Natural history of the infant gut microbiome and impact of antibiotic treatment on bacterial strain diversity and stability. Sci. Transl. Med..

[bib86] Zeevi D., Korem T., Zmora N., Israeli D., Rothschild D., Weinberger A., Ben-Yacov O., Lador D., Avnit-Sagi T., Lotan-Pompan M. (2015). Personalized nutrition by prediction of glycemic responses. Cell.

[bib87] Zhao S., Lieberman T.D., Poyet M., Kauffman K.M., Gibbons S.M., Groussin M., Xavier R.J., Alm E.J. (2019). Adaptive evolution within gut microbiomes of healthy people. Cell Host Microbe.

