# Supplementary material for: Dispersal strategies shape persistence and evolution of human gut bacteria
Source: Cell Host Microbe. 2021 Jul 14;29(7):1167–1176.e9. doi: 10.1016/j.chom.2021.05.008 (PMC8288446; doi:10.1016/j.chom.2021.05.008)
Supplement: Document S1. Figures S1–S4 [file mmc1.pdf]

Cell Host & Microbe, Volume *29*

Supplemental information

Dispersal strategies shape persistence and evolution of human gut bacteria

Falk Hildebrand, Toni I. Gossmann, Clémence Frioux, Ezgi Özkurt, Pernille Neve Myers, Pamela Ferretti, Michael Kuhn, Mohammad Bahram, Henrik Bjørn Nielsen, and Peer Bork

Supplementary Figures

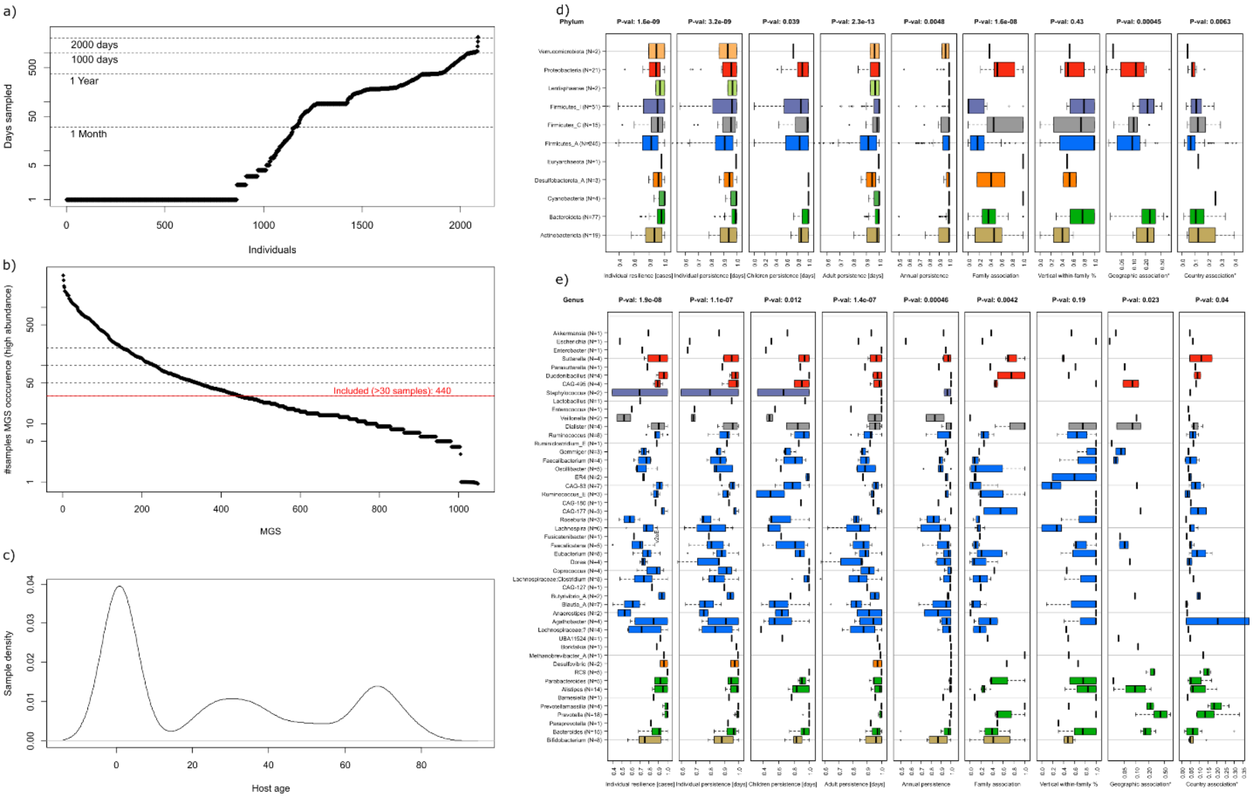

Supplementary Figure 1, Relates to Fig. 1: a) The time series analysed contains 2089 individuals and 5287 samples. The time span covered varies but is restricted to < 6 years. b) MGS included in the analysis of strain effects. Only MGS that were observed at sufficient abundance (see Methods) in at least 30 samples were included.

c) host age distribution was variable with about half of the samples originating from infant. c, d) Different forms of persistence are distinct among d) phyla and e) genera. Individual resilience and persistence, children and adult persistence are on an individual level, while family, country and geographic association approximates the ability of a species to be shared among (more or less) proximal individuals. Vertical within-family is the fraction of observed vertical transmissions, and some taxa like *Bifidobacteria* seem to be preferentially transferred from parent to child.

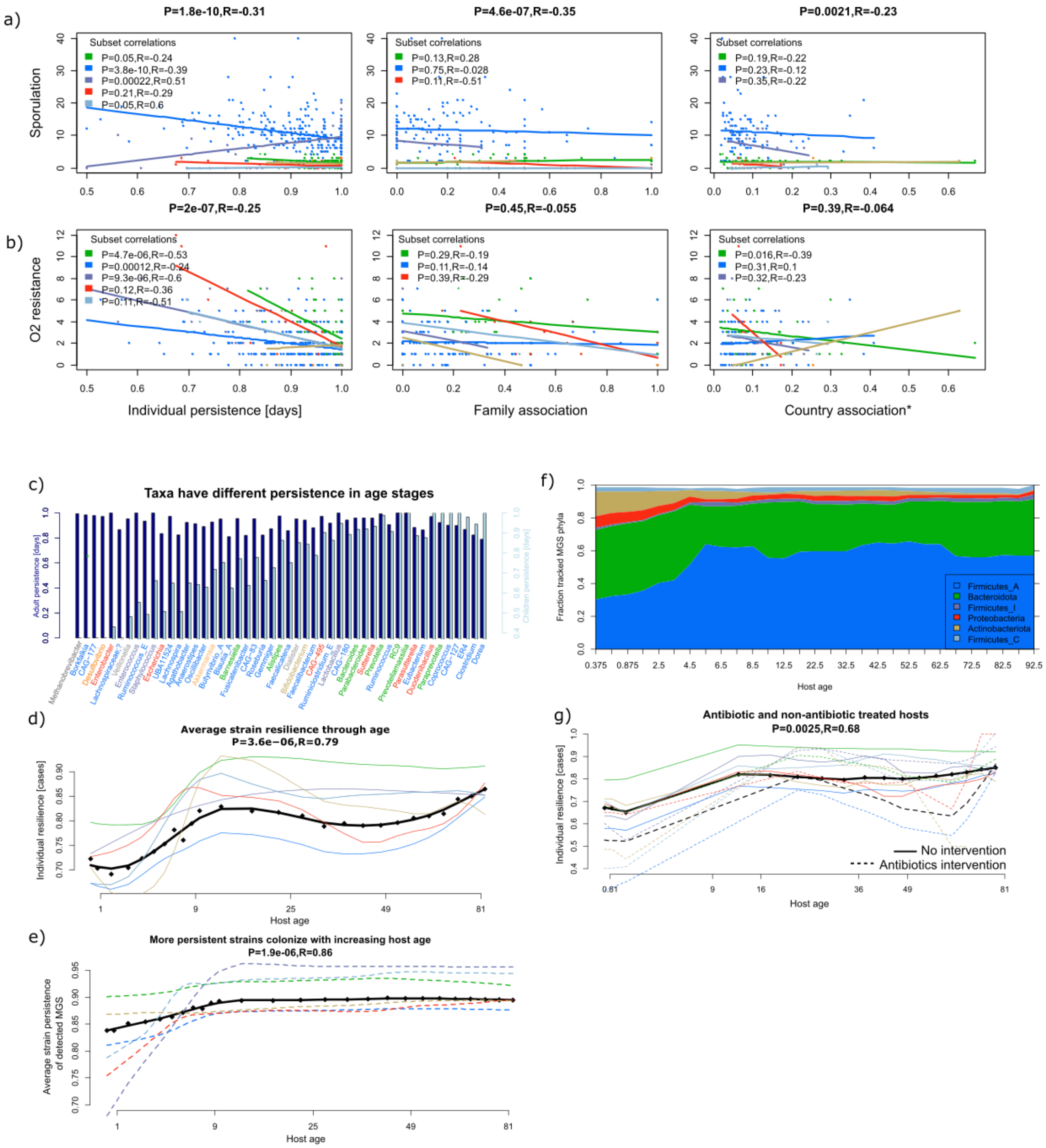

Supplementary Figure 2, Relates to Fig. 2: the amount of putative sporulation genes (a) and oxygen resistance related genes (b) found in each MGS is negatively related to a species ability to persist in their host or within families. For groups with >10 MGS data points, the correlation for subgroups are shown in the legend.

c) For the 40 most abundant strain genera and 10 selected taxa, the adult and infant individual persistence of strains (averaged per genera) are shown in contrasts. d) Dependent on host age, resilience and persistence of all taxa (black line) and phyla (dashed lines) changes. Data points (dots) were estimated with a 5-year spaced sliding window, that was finer spaced for children (at 0,0.25,0.5,0.75,1,2,3,4,5,6,7,8,9 years). All data points were summarized in a sliding window of size 5. e)

Considering only the average strain persistence calculated for each MGS across the complete dataset, we weighted each time-window by the detected MGS contribution to d). This showed that with increasing host age, we observed preferentially MGS that had a higher strain persistence, i.e. the species found in the adult microbiome have a higher average persistence, as measured on our dataset. This was also true for the main phyla (colour as in Fig 1b), with the possible exclusion of Bacteroidota. f) The taxonomic composition varied substantially during host age, even when limited to those MGS that were present at >0.1% abundance in at least two time-points per individual. g) Antibiotic treatment had mostly an impact on strain resilience. Dashed lines are persistence values for antibiotic treated hosts during the time-series, full lines for hosts where we knew that no antibiotic treatment was used. Colours represent phyla, as in Fig. 1b.

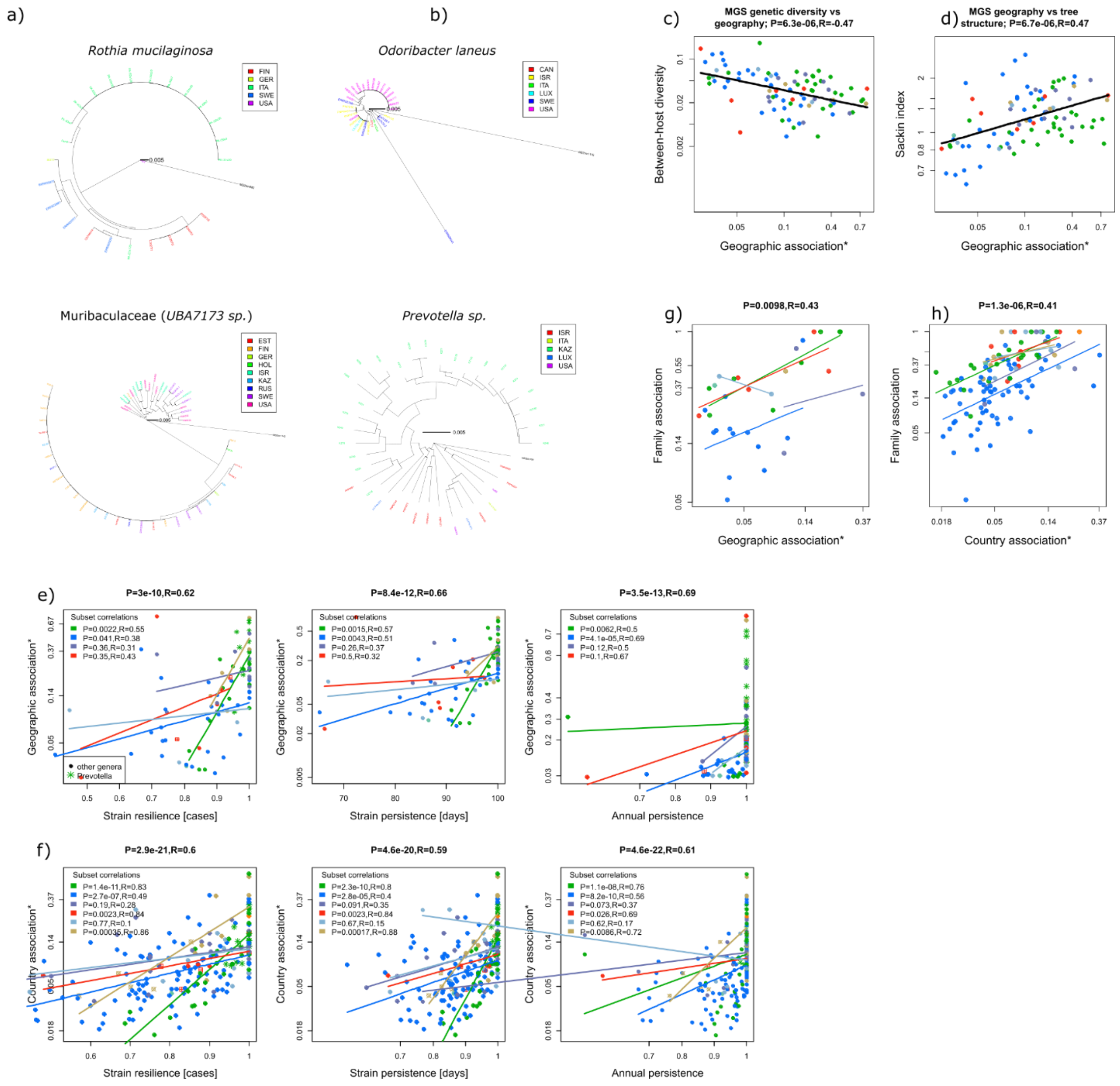

Supplementary Figure 3, Relates to Fig. 2: Local outbreaks are driving geographical stability
a,b)Examples of taxa with a high spatial stability, colors indicate the country of host origin. Only 1 sample per individual (or family if data available) was included.
c) between individual diversity (BID) is decreasing with higher spatial stability, indicating either that taxa with less diversity have a stronger spatial association. A local outbreak would consist mostly of clonal taxa with very similar genomes. d) Taxa with a higher spatial stability have a phylogeny with increased Sackin index, indicating a deviation from a balanced tree at 0. Colours represent phyla, as in Fig. 1b.
e,f) A MGS's ability to remain associated with its host is strongly correlated to it's e) country f) geographic association. Only MGS that were significantly associated to either countries of geographic coordinates were included, hence the sample number varies between Country and Geographic association.

g,h)Family association is significantly correlated to an MGS's g) geographic and h) country association This seems to be largely independent of taxonomic origin of an MGS. Only MGS that were significantly associated to either countries of geographic coordinates were included, hence the sample number varies between Country and Geographic association. Colours represent phyla, as in Fig. 1b.

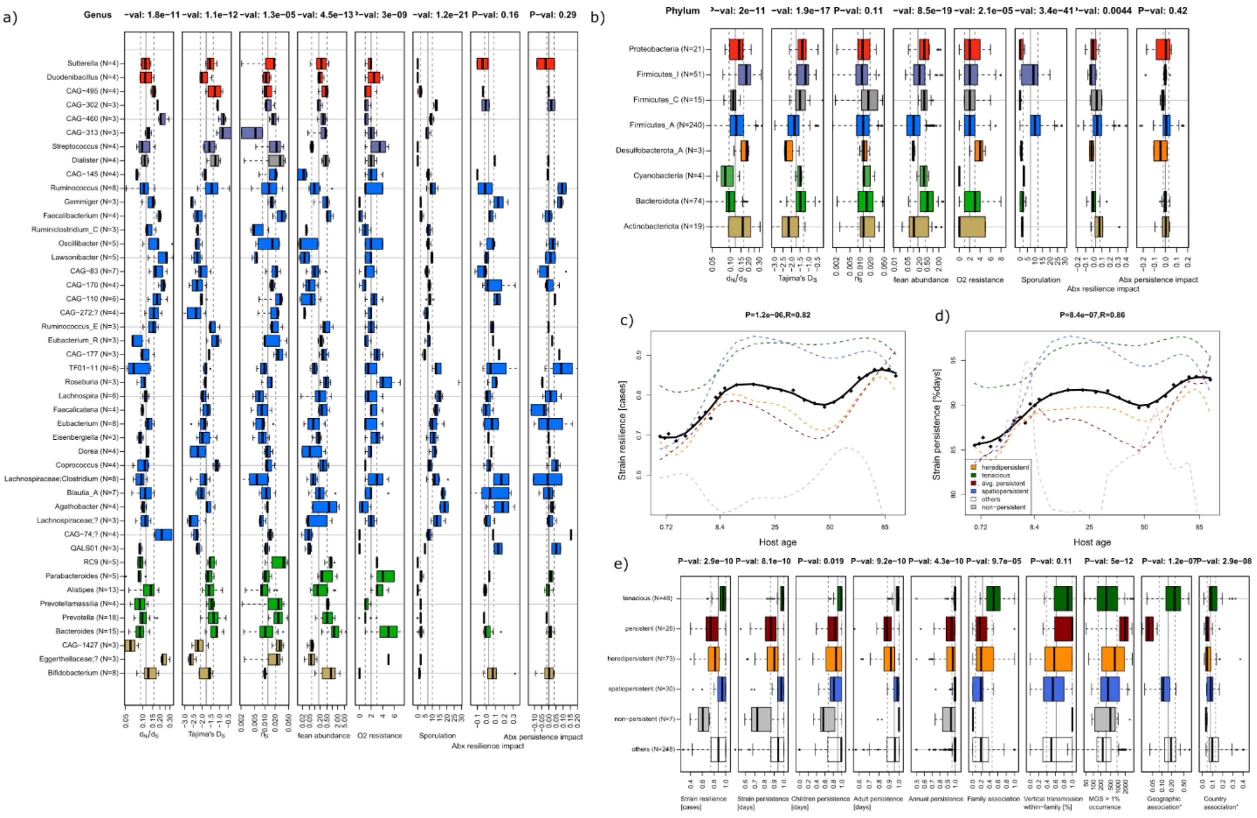

Supplementary Figure 4, Related to Fig. 3: a) Phylum and b) genus level summary boxplot denoting the distribution of population genetic parameters among MGS occurring in > 30 samples. Tajma's D and π_S were estimated from 20 randomly selected samples, restricted to 1 sample/family. O2 resistance and Sporulation are the genes found in each MGS, that can be assigned to oxygen stress resistance, sporulation or the (log10 transformed) ratio of resilience/persistence reduction in antibiotic treated individuals (see Methods).

c,d) Separating age-related c) resilience and d) persistence by species classified based on their dispersal strategy (heredipersistent, spatiopersistent, tenacious, avg. persistent or non-persistent, Fig. 4), reveals stark differences, with tenacious taxa having overall the highest host-association, spatiopersistent taxa being stronger host associated in adult host's, and heredipersistent and avg. persistent taxa having generally lowered persistence and resilience. Black line corresponds to average values across all taxa. Data points (dots) were estimated with a 5-year spaced sliding window, that was finer spaced for children (at 0,0.25,0.5,0.75,1,2,3,4,5,6,7,8,9 years). All data points were summarized in a sliding window of size 5. e) Strain resilience, persistence, persistence in child or adult samples, annual persistence, family association, the rate of vertical-to-horizontal persistence, Occurrence of species across all samples, geographic and country association were all compared among bacteria grouped in dispersal strategies.
